# VLM-guided retrieval augmented generation (RAG) for robot action prediction

**DOI:** 10.3389/fnbot.2026.1894674

**Published:** 2026-08-25

**Authors:** Boris Kuster, Nikola Marić, Fatima Aziz, Boshko Koloski, Andrej Gams, Aleš Ude

**Affiliations:** 1 Humanoid and Cognitive Robotics Laboratory, Department of Automatics, Biocybernetics and Robotics, Jožef Stefan Institute, Ljubljana, Slovenia; 2 Department of Knowledge Technologies, Jožef Stefan Institute, Ljubljana, Slovenia

**Keywords:** retrieval augmented generation, robot action prediction, robot learning in low-data regimes, robotic recycling of electronic waste, vision-language models

## Abstract

Reliable action prediction is essential for robotic electronic-waste disassembly, where device diversity and damage make preplanned sequences impractical. Vision-language models (VLMs) offer broad visual and semantic knowledge, but zero-shot predictions can be unreliable, while fine-tuning requires substantial task-specific data. We propose a VLM-guided retrieval-augmented generation (RAG) framework that crops task-relevant image regions, retrieves examples from a structured local database, re-ranks candidates, and expands the search when needed. We evaluated the framework on six types of smoke detectors and heat-cost allocators and compared the performance of three VLMs using zero-shot inference, single-example RAG, VLM-guided RAG, and task-specific fine-tuning. Cropping improved retrieval across all evaluated models, while VLM-guided re-ranking increased top-1 retrieval accuracy from 68.47% to 84.57-89.29%. Next action prediction accuracy reached 90.82-95.92% and significantly outperformed single-example RAG. RAG-based methods also outperformed the fine-tuned models. Median prediction latency was 8.95 s, although expanded searches were slower. Overall, VLM-guided RAG enables accurate, data-efficient adaptation to new device types without fine-tuning, at the cost of increased and variable latency.

## 1 Introduction

Standard methodologies for electronic waste recycling rely on a crush-and-separate approach, where devices are first crushed, followed by a physio-chemical separation of materials into useful categories. However, this approach may cause fires if dangerous components such as lithium-ion batteries are crushed (Simonic et al., 2024). Such components should therefore be removed before crushing each device. This requires their disassembly. Here a problem arises that in the recycling of electronic waste, it is necessary to deal with a wide variety of electronic items, be it from the same device family (e.g., smoke detectors) or even across different families of devices (e.g., smoke detectors, heat cost allocators, remote controls, etc.). Consequently, it is not feasible to plan every disassembly operation that may be necessary in advance. While devices may externally appear similar, their disassembly sequences can be significantly different. The correct disassembly sequence may also depend on the state of the device, as devices to be recycled may be partially damaged. Thus, it is necessary to plan robotic actions on the fly, or in other words, a recycling robot must be able to automatically predict the follow-up disassembly actions based on the current state of the device (Ude et al., 2025).

Vision is crucial to automate the disassembly of electronic devices. However, the available datasets are often small and costly to collect, which makes it difficult to train robust object detection and action prediction models capable of generalization (Abhimanyu et al., 2022). This is especially challenging in recycling, where robots must continuously handle new devices that were not present during training. These constraints motivate methods for effective robotic action prediction in low-data regimes, where fine-tuning large vision-language models is often impractical or ineffective, and where new examples should be incorporated with little or no additional training.

Recently, large Vision-Language Models (VLMs) have received increased attention in the field of robotics due to their substantial embedded world knowledge, which gives the possibility of considering general knowledge that is hard to incorporate or not available in classic planners and planning domains, e.g., in Planning Definition Domain Language (PDDL) (Ghallab et al., 1998). VLMs also have the ability to process one or more images and reason about objects and their relations (Li et al., 2024). A key advantage of VLMs (and LLMs) is the possibility of appending relevant examples to the input prompt, which is known as few-shot or in-context learning (Brown et al., 2020). While these relevant examples can be specified per-task by a programmer, a more general approach entails retrieving them dynamically from a database, based on the similarity of current inputs to the database elements. This approach is known as Retrieval-Augmented Generation (RAG) (Zhao et al., 2024) and allows VLM performance to be improved without computationally intensive fine-tuning, while also avoiding the possibility of catastrophic forgetting and hallucination (Towhidul et al., 2024).

**Figure 1:**
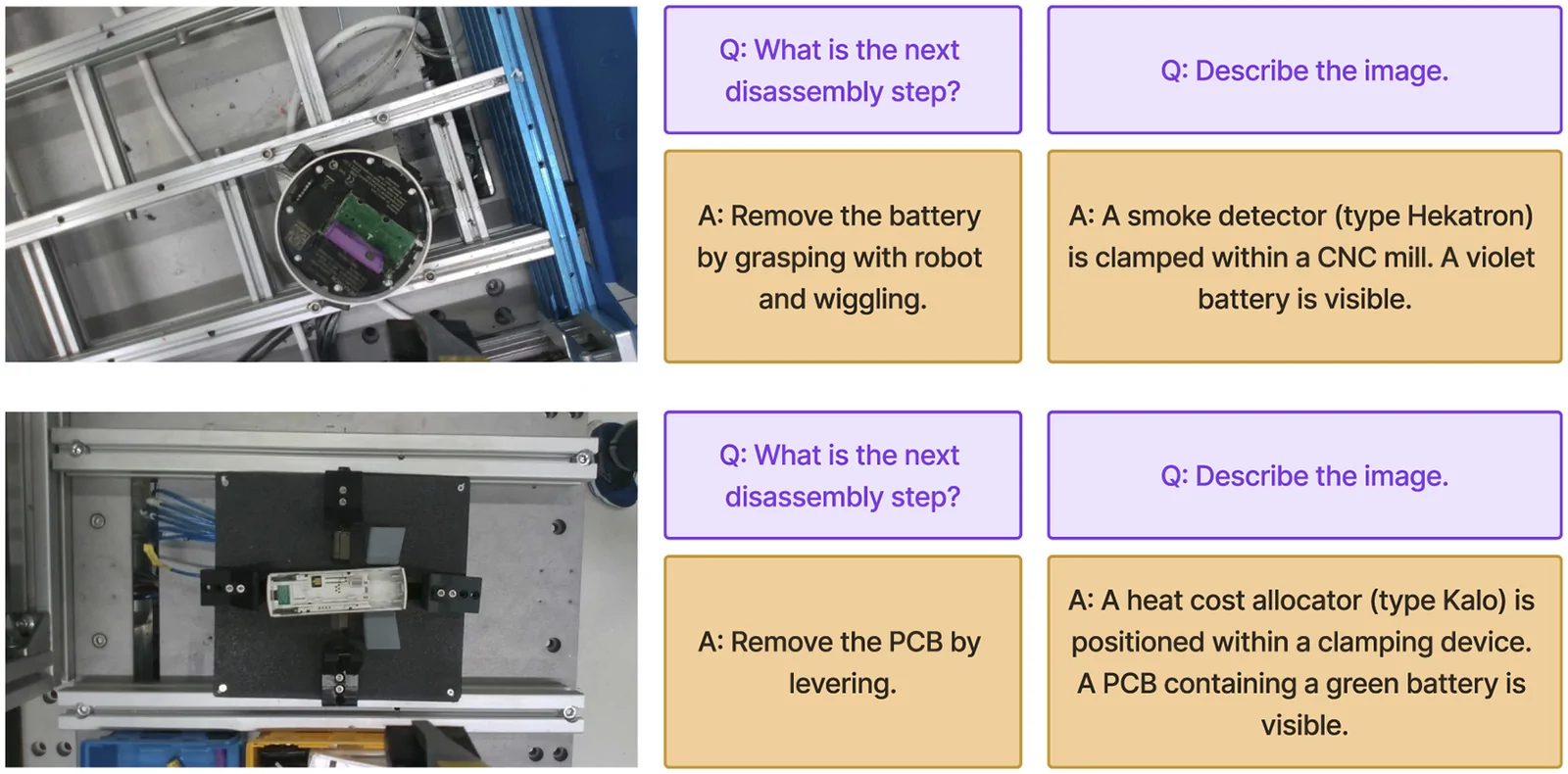
Example of two RAG data points containing an image and text - a set of question-answer pairs, used for disassembly action prediction.

The idea of RAG is to exploit a local database, where each data point can contain an image, text, and other modalities, or a combination thereof, as shown in Figure 1, all relevant in the context of the desired task. These data points are then embedded into the vector space by means of a modality-specific embedding model, which is typically a much smaller model than a VLM (Radford et al., 2021). When a downstream model, e.g. a VLM, is requested to perform inference on a novel data point, elements of the input data point, e.g. the input image, are utilized by the embedding model, relevant examples (image & corresponding text) are retrieved from the database, and added to the VLM prompt. This approach increases the prediction accuracy of VLMs (Mortaheb et al., 2025). Especially for smaller datasets, RAG can be more effective than fine-tuning (Lakatos et al., 2025). Some important advantages of using a RAG system are that adding data points does not require retraining, the performance of the downstream model can be increased, and the database can also be used as a fine-tuning dataset if necessary.

In a baseline RAG approach, only the most similar data point is retrieved from the database and added to the VLM prompt. This has several potential downsides:

- The database may lack relevant examples and including irrelevant data points may decrease prediction accuracy,
- the most similar data point in the embedding vector space may not be the most representative, since embedding models are not perfectly accurate,
- the input query may contain distractor elements, e.g., an image where the relevant object only takes up a small portion of the input image, decreasing the embedding quality, and
- in case of text-based retrieval, embedding models may struggle on unstructured or longer text segments.

**Figure 2:**
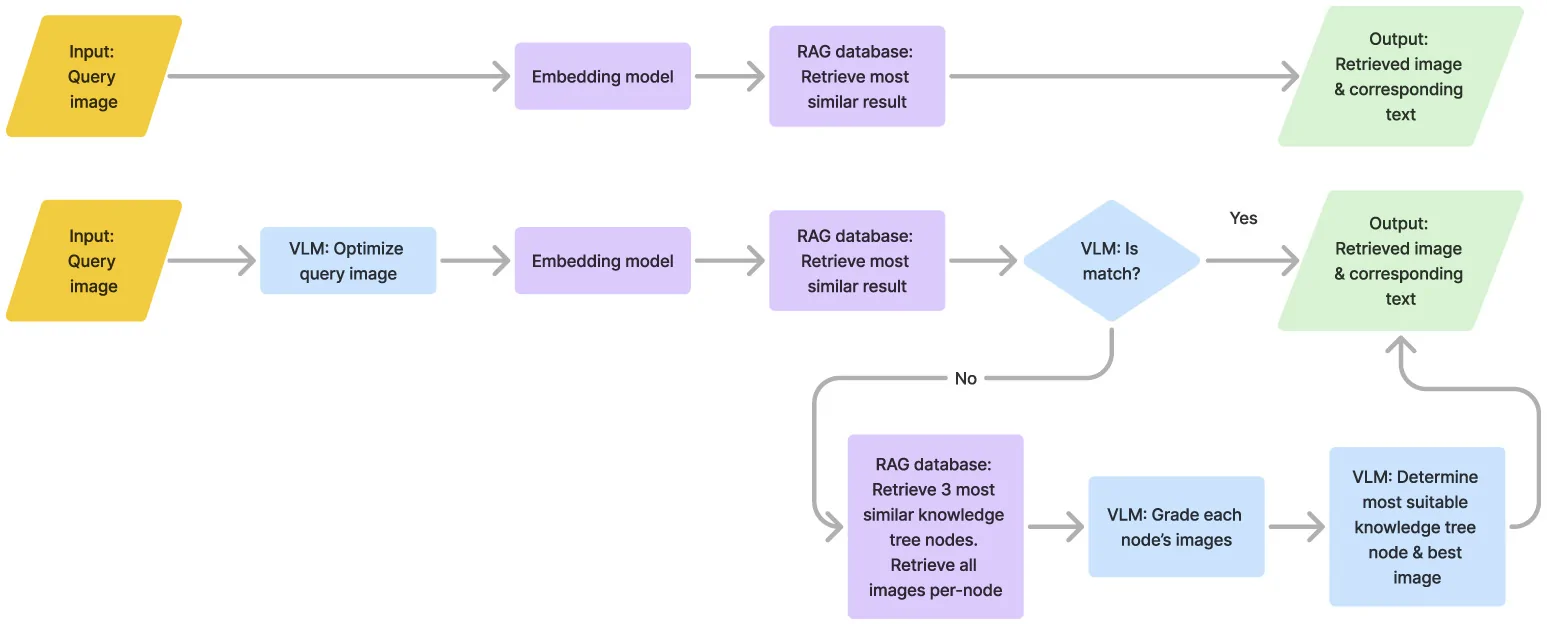
Comparison of baseline RAG **(top)** with the proposed VLM-guided RAG approach **(bottom)**. The additional elements are shown in blue.

**Figure 3:**
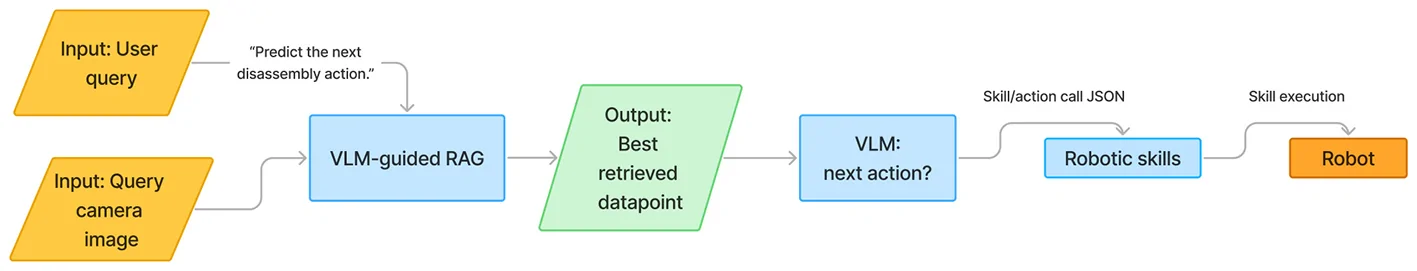
The final action prediction & execution pipeline.

To mitigate these issues, we propose an algorithm called VLM-guided RAG, in which a VLM is used to refine (crop) the input query image and the images stored in the RAG database, re-evaluate the retrieved results, and iteratively expand retrieval until the VLM determines that a suitable data point has been found or a preset time limit is reached. This process, shown in Figure 2, yields an informative example that can be passed to downstream models. In robotics, especially in dynamic applications such as recycling, this can improve the performance of VLMs for next-action prediction and related tasks. The full action prediction pipeline is shown in Figure 3. We demonstrate the method on action prediction for the disassembly of waste electronics. Such AI-based approaches can significantly reduce the programming workload required to adapt robotic workcells to new applications (Gaspar et al., 2020). To evaluate effectiveness, we compare VLM-guided RAG against several baselines: a standard RAG approach that retrieves a single most similar element and a non-RAG (zero-shot) setting in which the VLM receives no additional task-specific examples. Each method is evaluated with both open-source and commercial VLMs, allowing us to assess performance across model families as well as retrieval strategies.

## 2 Materials and methods

Action prediction in robotics is a key challenge for increasing the automation of robotic workcells and reducing the programming effort required to deploy them in new tasks, ultimately enabling robots to perform a broader and more diverse range of tasks. Traditional approaches have relied on neural-network models such as multi-layer perceptrons (MLPs), recurrent neural networks (RNNs), and long short-term memory networks (LSTMs) (Mavsar et al., 2021). While effective in specific settings, such models often suffer from poor generalization and typically require task-specific training.

Recently, vision-language models (VLMs) have shown impressive results, offering a promising direction for next-action prediction in robotic workcells thanks to their strong generalization, large-scale pre-training, and ability to interpret visual inputs (Li et al., 2025). However, VLMs are rarely pre-trained on data that closely matches real-world robotic task contexts. Fine-tuning them with task-specific data therefore remains a challenge due to the limited size of available action prediction datasets and substantial computational costs required to train larger models, which generally outperform their smaller counterparts (Kaplan et al., 2020).

To improve the accuracy of action prediction without fine-tuning a VLM, a practical solution is to leverage a local database of examples via RAG. This approach enables the model to retrieve and reference relevant examples during inference, bridging the gap between general-purpose VLM knowledge and domain-specific information. However, the way in which baseline RAG is commonly utilized, i.e., to retrieve a single most similar example, is often suboptimal (Zheng et al., 2025). In addition, retrieval quality depends heavily on the accuracy of the embedding models, which are not perfectly reliable and are usually much smaller than the VLM.

To improve both the retrieval and action prediction performance, we propose a **VLM-guided RAG** system, which consists of the following main components:

1. RAG image query optimization (see Section 2.2)
  A VLM improves the relevance of the query image by iteratively cropping irrelevant regions, providing the embedding model with a more focused visual input and improving the retrieval performance. The same procedure is also applied to refine images stored in the RAG database.
2. Similarity-based retrieval (see Section 2.3)
  The cropped query image is encoded using an image embedding and the system retrieves most similar image-text data points from a vector database and a structured knowledge tree.
3. VLM-guided re-ranking and search expansion (see Section 2.4)
  A VLM iteratively re-ranks the retrieved candidates by jointly evaluating the query image and the retrieved candidate's image and text. The search is expanded when necessary, yielding a substantial improvement in final retrieval accuracy compared to standard RAG.

The proposed approach allows us to leverage the high-level visual and text reasoning capabilities of VLMs to compensate for the limitations of smaller image-embedding models. A comparison between the baseline RAG pipeline and our proposed approach is shown in Figure 2.

To evaluate VLM prediction performance in the zero-shot setting as well as under the proposed RAG-based approaches, we collected an action prediction dataset originating in robotic disassembly of waste electronics, described in Section 3.1. This dataset encompasses a variety of tasks and action possibilities.

### 2.1 VLM tool calling

To ensure that the VLM produces the specific type of output required at each stage of our pipeline (e.g., image query optimization, VLM-guided re-ranking and search expansion) structured in a proper format, we use a method known as tool calling (Gao et al., 2025). In tool calling, the programmer (i.e., us) defines a set of “tools” the VLM is allowed to use. Each tool specifies:

1. a name (e.g., SetGridSize),
2. a tool description,
3. the parameters it accepts (e.g., gridSize), and
4. the type of each parameter (e.g., integer, string).

These tool definitions are provided to the VLM prior to response generation. Guided decoding (Willard and Louf, 2023) is then used to constrain the model's output to exactly match the predefined schema. This way we always obtain a valid, structured, machine-readable response. The approach also enables real-time adaptability, since no model fine-tuning is required in the event that tool definitions change or new tools are introduced. We apply this approach for the VLM-guided RAG pipeline and the next-action prediction, so that every VLM output is not only semantically correct but also structured appropriately for the subsequent steps in the workflow.

### 2.2 RAG image query optimization

The performance of a RAG system is highly sensitive to the quality of its input query (in our case, an image). Prior work has focused on optimizing text-based RAG (Gao et al., 2022) and on optimizing vision-based RAG for document retrieval (Yu et al., 2025), whereas query optimization for vision-based RAG in robotic settings remains comparatively unexplored.

To improve the quality and retrieval performance of RAG, we propose an input image optimization (cropping) pipeline that uses a VLM to increase the relevance of the query image. Since the raw query image may contain irrelevant content, the VLM is tasked with selecting a region of interest (ROI) and cropping the image, typically over multiple iterations. The resulting cropped image is passed to the image encoder, producing a more informative vector embedding and, consequently, more relevant retrieved examples. The same cropping strategy is used when constructing or incrementally updating the RAG database, as it is crucial that both database images and query images contain maximally relevant visual information for the task. Without consistent cropping on both sides, semantic mismatches may occur. For example, a database image may show an entire desk with a small target workpiece in the background, while the cropped query image contains only the workpiece itself. Unless all images are cropped, such discrepancies can degrade retrieval because the embeddings reflect different visual focus and context.

Although some recent VLMs can output bounding boxes [e.g., Qwen3-VL (Wang et al., 2024)], the object localization accuracy is still unreliable, particularly in the case of novel, out of distribution objects not present in the dataset. However, recent work has shown that VLMs can benefit from simple visual scaffolds and annotations (Lei et al., 2024). In particular, SCAFFOLD prompting overlays a dot matrix on the image as visual anchors and uses multi-dimensional coordinates in the text prompt as positional references.

Based on these observations, we designed and evaluated two iterative cropping strategies—grid-based and bounding-box-based – to enable the VLM to focus on the relevant image region. At each iteration, the VLM is provided with (i) the full-size query image, (ii) the current cropped view, and (iii) a textual instruction (e.g., “Crop the image so the electronic device is centered. Keep relevant neighboring information, such as the fixture or housing in which the device may be mounted.”). Since our dataset consists of electronic devices, we use a single, task-specific prompt suitable for all electronic devices in our dataset. However, the prompt can be adapted for other use cases. The model then proposes an updated crop while preserving task-relevant context (e.g., a clamp or holder). To ensure reliably parsable output, the VLM is constrained to produce tool calls with predefined parameters. The final cropped image is then embedded and used for similarity-based retrieval in the RAG pipeline.

#### 2.2.1 Grid-based cropping

Grid-based image cropping is implemented by overlaying the input query image with a grid and assigning a single integer to denote the ID of each grid rectangle.

We define the following tools that the VLM is allowed to use:

- Crop: Crop the image by specifying the top-left and bottom-right grid cell indices, and extract the corresponding rectangular region,
- SetGridSize: Change the grid resolution by setting the number of rows and columns,
- Reset: Reset the current cropped view to the original (full) image, and
- Finish: Conclude the task and return the final cropped image together with its bounding box.

**Figure 4:**
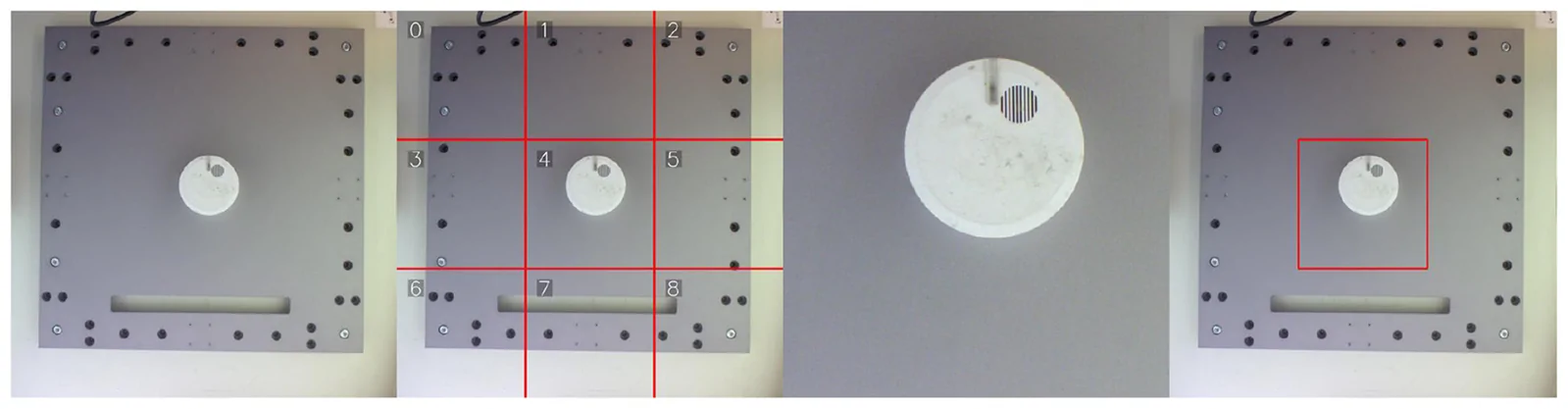
The input query image, its grid-annotated image, the final cropping result, and the cropped area shown in the original image.

In an iterative procedure, shown in Algorithm 1, the VLM repeatedly selects a tool and its arguments until the final crop of the image is achieved, as determined by the VLM. If the grid lines intersect with the relevant image parts, the VLM can adjust the grid resolution by changing the number of rows and columns. Cropping is performed by specifying the IDs of the top-left and bottom-right grid elements, which define the rectangular ROI. Since the original input query image is always included in the prompt, the VLM can also reset the current cropped view to the full image if an intermediate crop is unsatisfactory. Finally, the VLM is instructed to select the *finish* tool when the image is sufficiently cropped. We impose a maximum number of iterations to guarantee termination. Figure 4 shows an example input image, its grid-annotated version, and the resulting final crop.

Algorithm 1 Grid-based RAG image query optimization using a VLM. 
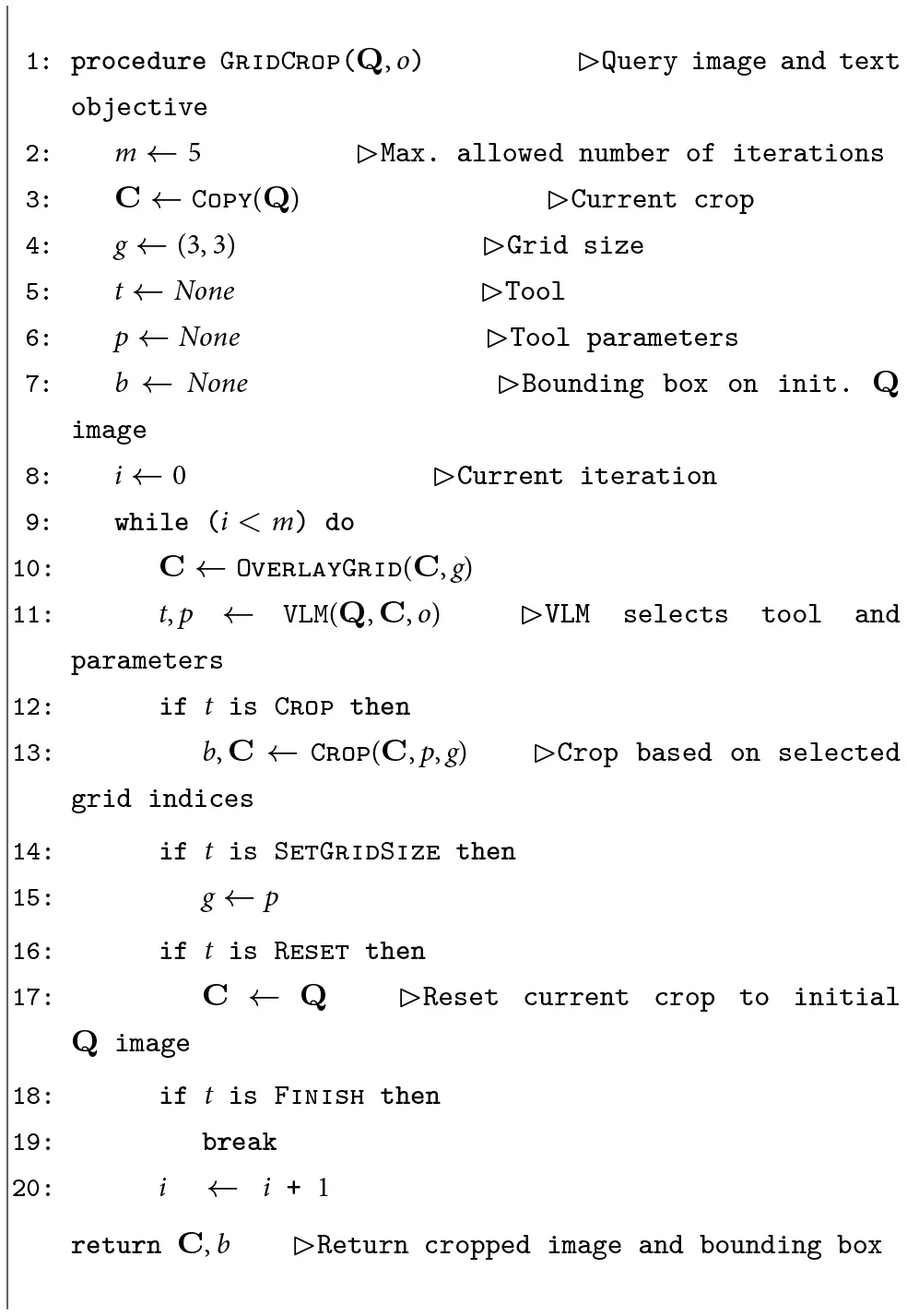

During the iterative cropping process, the image is downscaled to a predefined resolution to reduce VLM inference time. The original high-resolution image is retained, and the final crop is extracted from it, ensuring that the resulting cropped image preserves full detail. Compared to the grid-based approach, iterative bounding box prediction enables finer, more flexible localization, but it also relies more heavily on the VLM's spatial localization accuracy and can be less robust.

#### 2.2.2 Bounding box based cropping

The second approach we propose is iterative bounding box prediction. In this approach, the VLM outputs normalized top left bounding box coordinates together with the normalized bounding box width and height. Compared to the grid-based method, this representation provides finer-grained and more flexible localization, which we hypothesize will yield improved performance. The VLM is provided with the following tools:

- SetBoundingBoxParameters: Specify a bounding box using normalized top left (*x, y*) coordinates, width, and height.
- Finish: Conclude the task and return the final cropped image extracted from the bounding box.

This iterative procedure allows the VLM to progressively refine the bounding box based on the input image and the currently selected bounding box. As with the grid-based approach, we impose a maximum number of iterations to guarantee termination. The procedure is summarized in Algorithm 2.

Algorithm 2 Bounding-box-based RAG image query optimization using a VLM. 
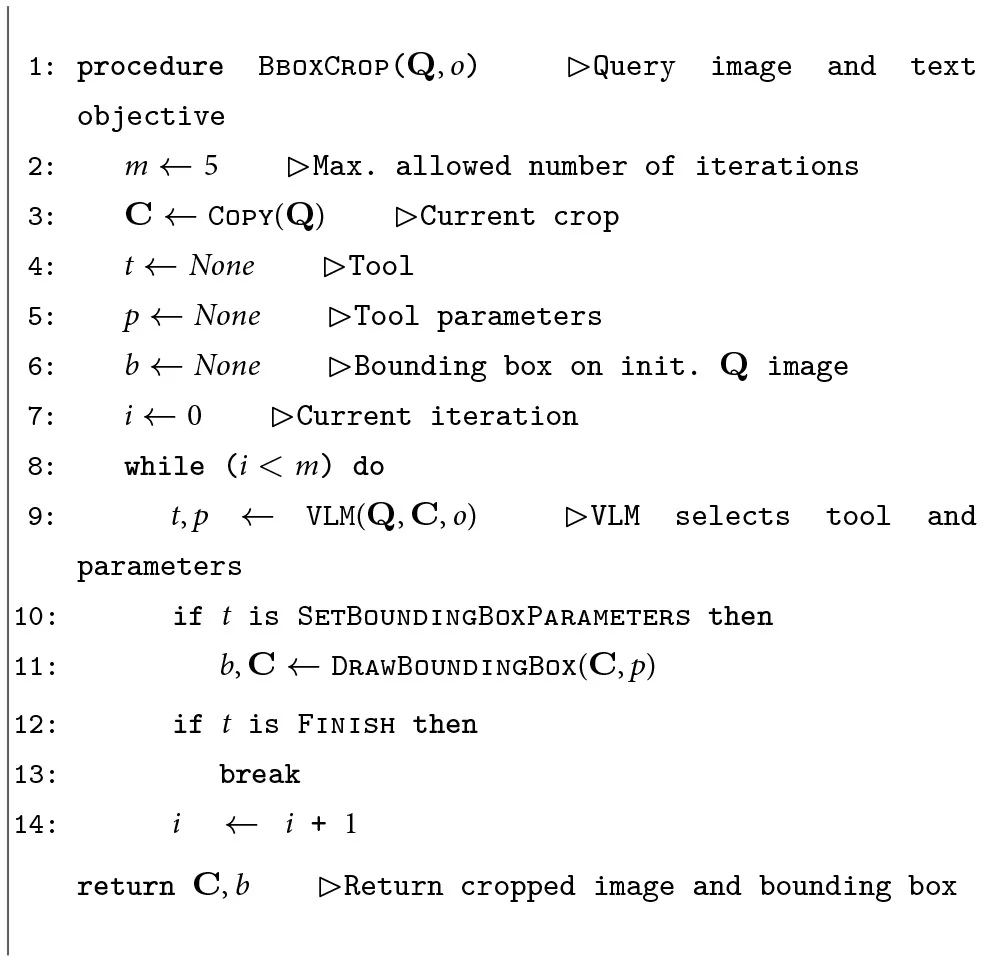

### 2.3 RAG knowledge base and lookup

Our RAG knowledge base combines two key components: a structured knowledge tree and a vector database. These enable efficient and accurate multimodal retrieval for downstream tasks, e.g., disassembly action prediction. The vector database facilitates similarity-based image retrieval, allowing the system to find visually relevant data points based on an input query image. Meanwhile, the knowledge tree provides a hierarchical organization of data points (devices and their disassembly sequences), linking each image to structured textual information and also enabling an overview of device types present in the database.

#### 2.3.1 Similarity-based image retrieval

In our setting, each data point in the knowledge base contains both an image and the associated text. Therefore, when we perform image-based search, the corresponding text is also retrieved, which assists the VLM in making accurate next-action predictions. The text always includes the device name and type. This information serves as entry point for follow-up lookups in the structured knowledge tree, which enables the system to retrieve full disassembly sequences and other device-specific information.

To select candidate images (and the corresponding text) from a knowledge base based on an input query image, an image embedding model *emb* is utilized. Some commonly used image embedding models include *CLIP* (Radford et al., 2021), *ResNet* (He et al., 2015), and *VisionTransformer* (Dosovitskiy et al., 2021). While *ResNet* and *VisionTransformer* are primarily used as image classification models, they can also produce embedding vectors for images.

Embedding models are preferred over direct image-to-image comparison, e.g., pixel-wise distances, because they map images into a compact semantic feature space, which is robust to irrelevant variations such as illumination changes, viewpoint, background clutter, and minor occlusions. This makes similarity search more meaningful as it captures visual/semantic relatedness rather than raw pixel similarity. It is also more computationally efficient, since retrieval is reduced to comparing fixed-length vectors instead of high-dimensional image data.

Formally, the embedding model *emb* with parameters θ calculates an embedding vector **x** for the query image *I*, which is then compared to vectors *x*_*j*_ generated from the database images *I*_*j*_, *j* = 1, …, *N*, using cosine similarity. Here, *N* denotes the number of data points in the knowledge base. Cosine similarity is defined as


$$\begin{array}{l} \text{x}=\text{em}{\text{b}}_{\theta }\left(I\right), \end{array}$$ (1)

$$\begin{array}{l} {\text{x}}_{j}=\text{em}{\text{b}}_{\theta }\left(I_{j}\right), \end{array}$$ (2)

$$\begin{array}{l} s_{j}=\frac{x\cdot x_{j}}{||x||\;||x_{j}||}. \end{array}$$ (3)

**Figure 5:**
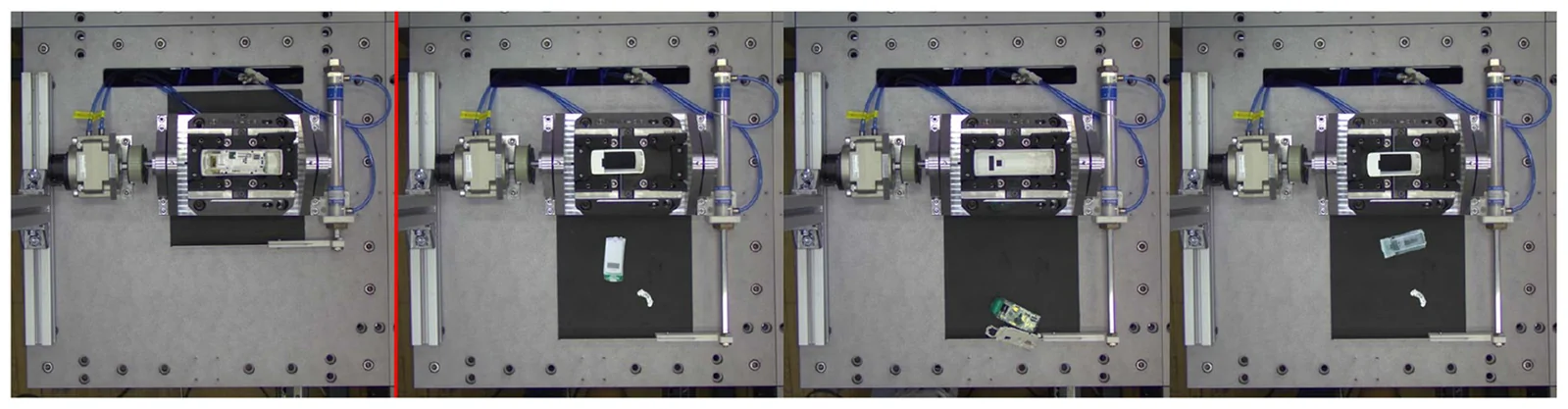
Example results retrieved by RAG, where the left-most image is the query.

Top *n* most similar database elements are returned, where the number of candidates *n* is specified by the user. An example of a query image and the corresponding retrieved images is shown in Figure 5.

#### 2.3.2 Knowledge tree

A downside of a standard vector database is that data points cannot be hierarchically structured. For robotic disassembly action prediction, however, it is useful to organize devices into families and to keep all disassembly steps for a specific device grouped together. To this end, we utilize a knowledge tree which provides a hierarchical organization of devices and their disassembly sequences. This structure also enables semantic queries over the dataset, such as retrieving available device families or all devices belonging to a specific device family (e.g., all smoke detector variations), although such queries are not explicitly used in our method.

**Figure 6:**
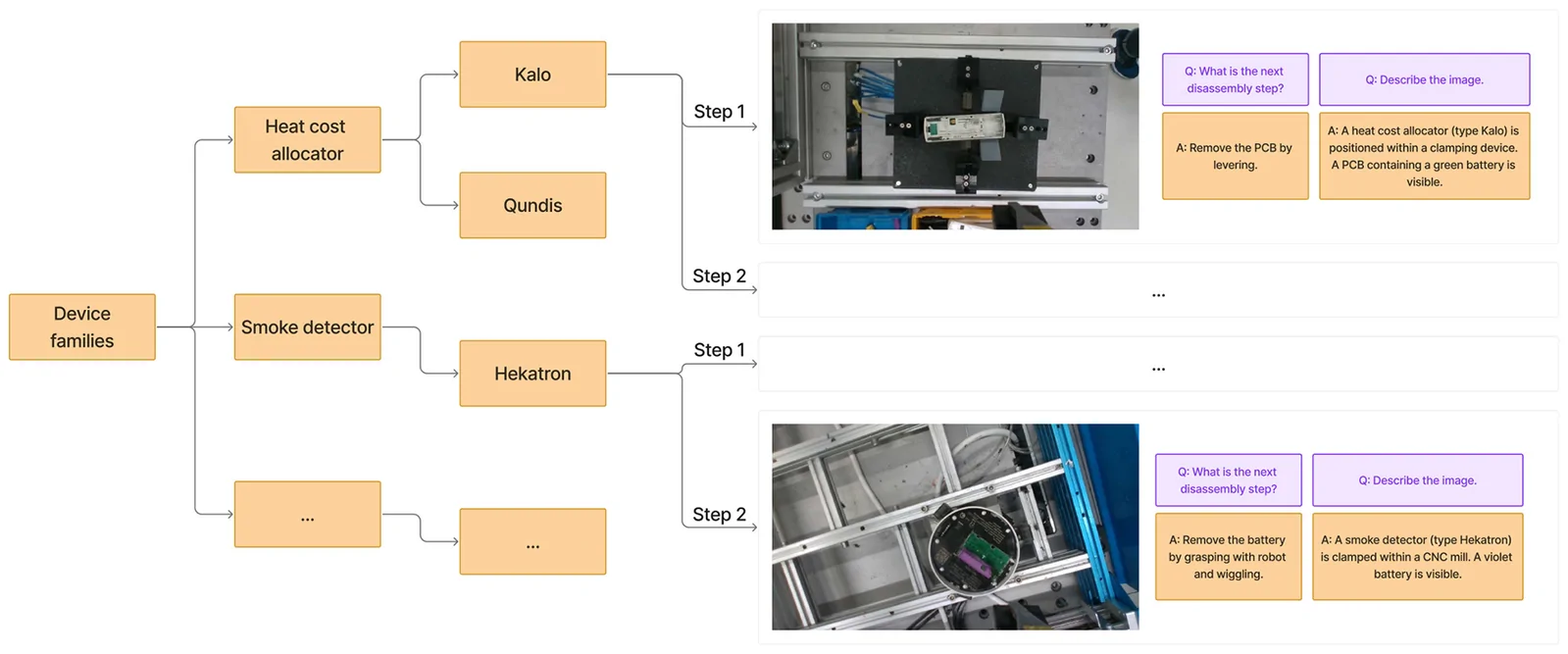
Database structure showing the knowledge tree nodes containing relevant text and embedded images of devices' disassembly steps.

Each knowledge tree's leaf node represents a particular device (e.g., smoke detector Kalo), while the parent node represents the device family (e.g., smoke detector). For each disassembly step, an image along with the corresponding ground truth text is stored within the device leaf node. The corresponding text contains all relevant information for action prediction, such as information about the device name and type, next required disassembly action, and the remaining sequence of disassembly steps. The corresponding images are embedded in the vector database and connected to the knowledge tree. Therefore, when an image is returned using a vector search, the leaf name is also included in the retrieved text and metadata, allowing for retrieval of all sequential disassembly steps (images & texts) for a particular device. Figure 6 shows an example of the knowledge tree structure.

### 2.4 VLM-guided re-ranking and search expansion

In many cases, particularly for robotic action prediction, retrieving only the most similar data point is insufficient due to imperfect embedding model performance. While the RAG database allows for retrieving an arbitrary number of data points (i.e., images & corresponding text), simply appending several data points to the VLM prompt is ineffective for the following reasons:

- the prediction latency is significantly increased when processing multiple high-resolution images, and
- model performance is known to degrade with longer prompt lengths, a problem known as “context stuffing” (Du et al., 2025). In case of multiple images, the VLM may lose its focus on the query image.

For these reasons, it is preferable to first conduct a more detailed retrieval step to ensure that the most representative example data point is correctly identified in the database. Since VLM inference over multiple images is slow, we need to be careful to preserve the computational efficiency of baseline RAG and avoid overly increasing prediction latency.

To achieve these objectives, we developed a novel retrieval algorithm called VLM-guided re-ranking and search expansion. The first step of our algorithm is to re-evaluate the most-similar RAG-retrieved candidate image with respect to the query image. The VLM is instructed to provide a positive or negative score to the image, based on (i) location similarity (e.g., CNC machine, flat table, ...), (ii) object shape similarity, (iii) similarity of internal electronic components, and (iv) ground-truth consistency with the preceding disassembly steps for both the candidate and the query images. If the VLM confirms the image, the database image and the corresponding text are directly passed to the action prediction VLM, which ensures minimal additional processing time. For this step, the VLM is provided with the following tool:

- VLM.BoolGradeImage: Compare the top-1 RAG-retrieved candidate image with the query image and return a positive or negative score, together with reasoning.

If, however, the VLM rejects the obtained candidate image, we retrieve *n* = 15 most-similar images from the RAG database. Then, we determine which leaf nodes (i.e., devices) the images belong to and de-duplicate the leaves, keeping the *m* = 3 most relevant knowledge tree leaves. These parameters may be modified based on the complexity of the database and the task. As mentioned, each leaf contains the images of a single device during all sequential disassembly steps, along with ground-truth text describing the previously executed actions, the device model name (e.g., “Fumonic”) and class (e.g., “smoke detector”), and the ground truth next disassembly action.

For each retrieved leaf and its images, the VLM grades each of the images sequentially, before providing a final score (0–10) of the leaf (i.e., device) similarity to the query image, and a summary of findings. For each leaf, the image or images with the highest score are stored together with the corresponding text; in cases where multiple images obtain the same highest score, all tied images are retained. The procedure for grading a single leaf is shown in Algorithm 3. Optionally, the VLM can extend the search to a maximum of *m* = 5 leaves.

Algorithm 3 VLM-guided leaf grading. 
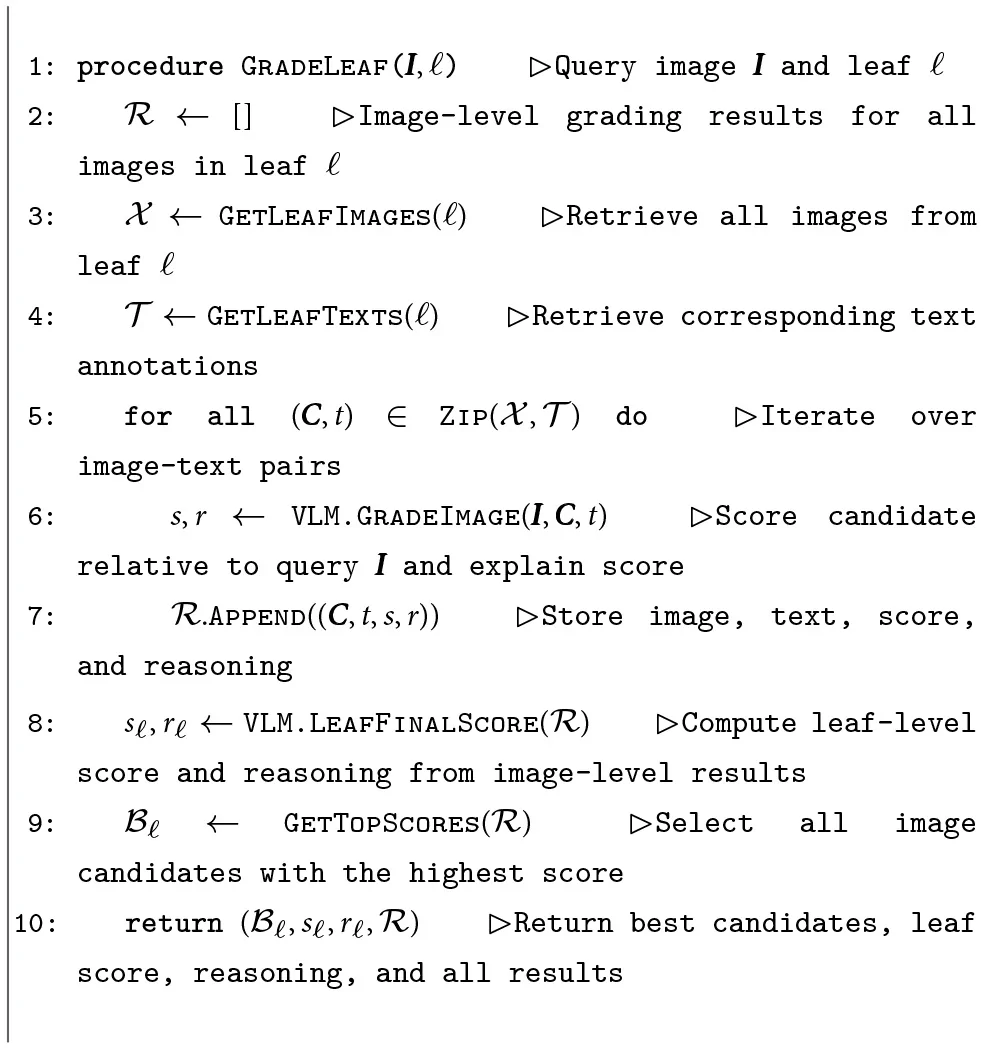

In this step, the VLM is provided with the following tools:

- VLM.GradeImage: Provide a 0–10 score of a candidate image, along with reasoning.
- VLM.LeafFinalScore: Provide a 0–10 score of a leaf node, based on combined textual reasoning about a particular leaf's images.
- VLM.ExpandSearch: Expand the search by retrieving all images & text of the next most-similar knowledge tree leaf.

In case of highest score collisions (i.e., when two images, either from the same or different leaf nodes, are assigned the same highest score), an iterative elimination process is performed—VLM-guided three-image tie resolution, shown in Algorithm 4. This process involves the query image and the set of candidate images that are tied for the highest score. In each iteration, two candidate images are selected and the VLM is used to determine the better candidate in relation to the query image. The lower-scoring image is eliminated from the set. This is repeated with the remaining candidates until only one image, which is considered the most similar to the query image, remains. For this procedure, the VLM is provided with the following tool:

- VLM.CompareImages: Compare two candidate images and their corresponding ground-truth text, with respect to the query image and return the candidate that is more similar to the query image.

Algorithm 4 VLM-guided three-image tie resolution. 
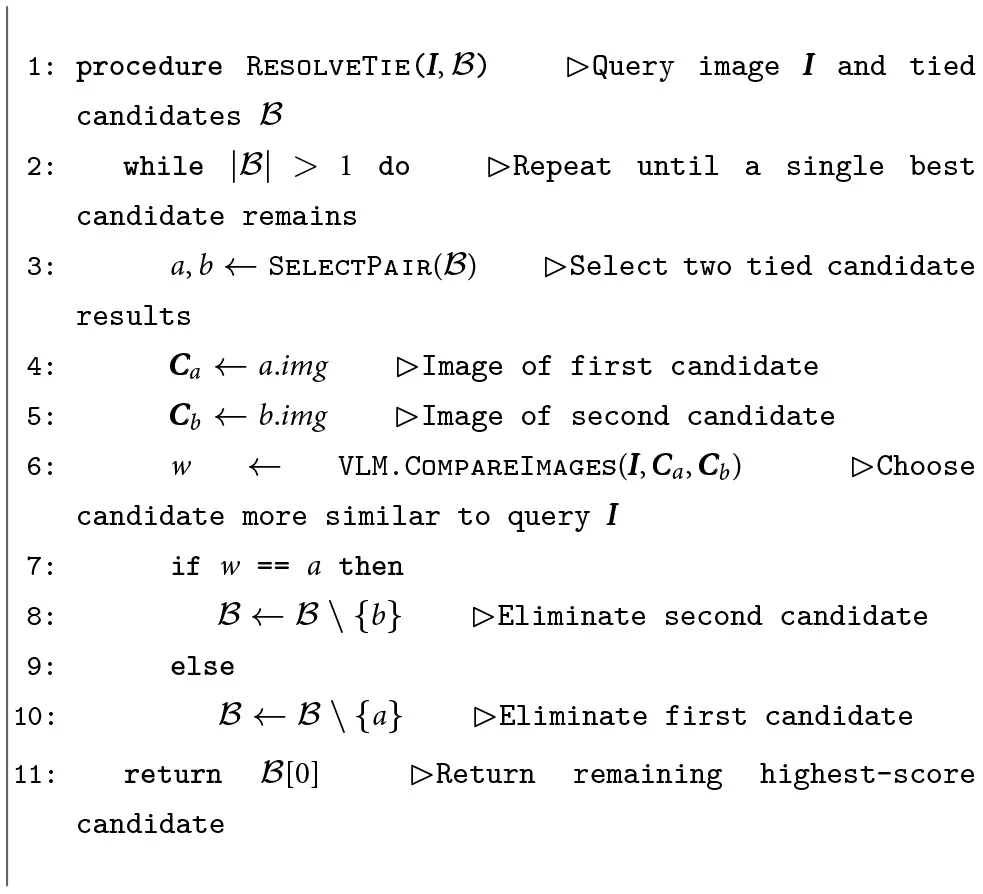

The final output of our VLM-guided re-ranking and search expansion algorithm, shown in Algorithm 5, is the single most relevant retrieved image and the corresponding text. Additionally, the VLM is asked to output reasoning for the steps of the grading process to improve its performance using the Chain-of-thought methodology (Wei et al., 2023) and also allow for failure-case analysis. The retrieved image & text are then passed to the VLM in downstream tasks, such as next-action prediction in dynamic robotic tasks. This search process increases retrieval accuracy over baseline RAG. A comparison of baseline RAG and our VLM-guided RAG approach is shown in Figure 2. Note that the optional search expansion step is omitted from the flow chart for visual clarity.

Algorithm 5 VLM-guided re-ranking and search expansion. 
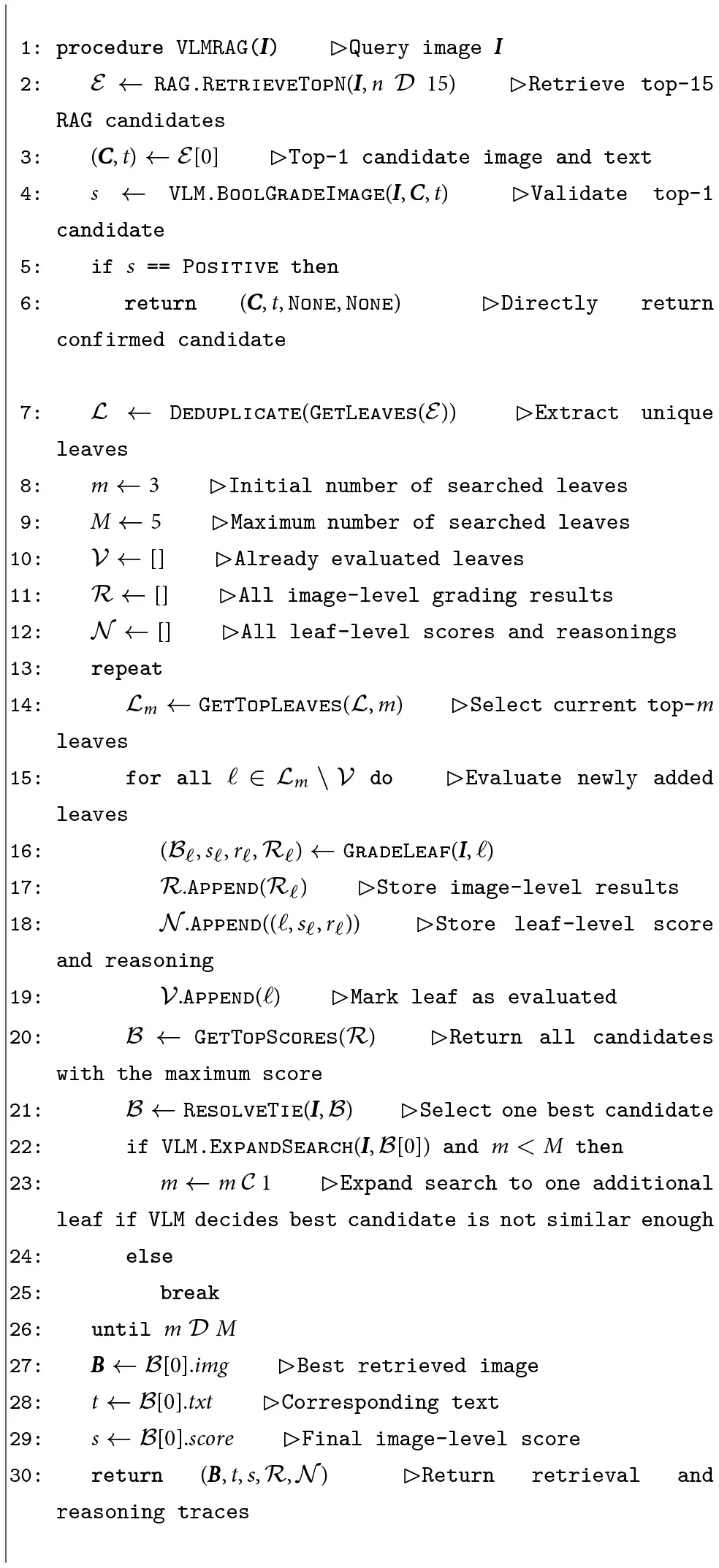

## 3 Experimental evaluation

This section describes the experimental setup used to evaluate the different system components. Firstly, the dataset and action prediction tasks are described in Section 3.1. Statistical tests used to determine significance are discussed in Section 3.2.1. The evaluation metrics for RAG image query optimization are presented in Section 3.2.2. The metrics used to evaluate the performance of different image embedding models are shown in Section 3.2.3. Lastly, the metrics used for evaluating action prediction performance and a summary of evaluated models and methods are shown in Section 3.3.

### 3.1 Dataset and tasks

**Figure 7:**
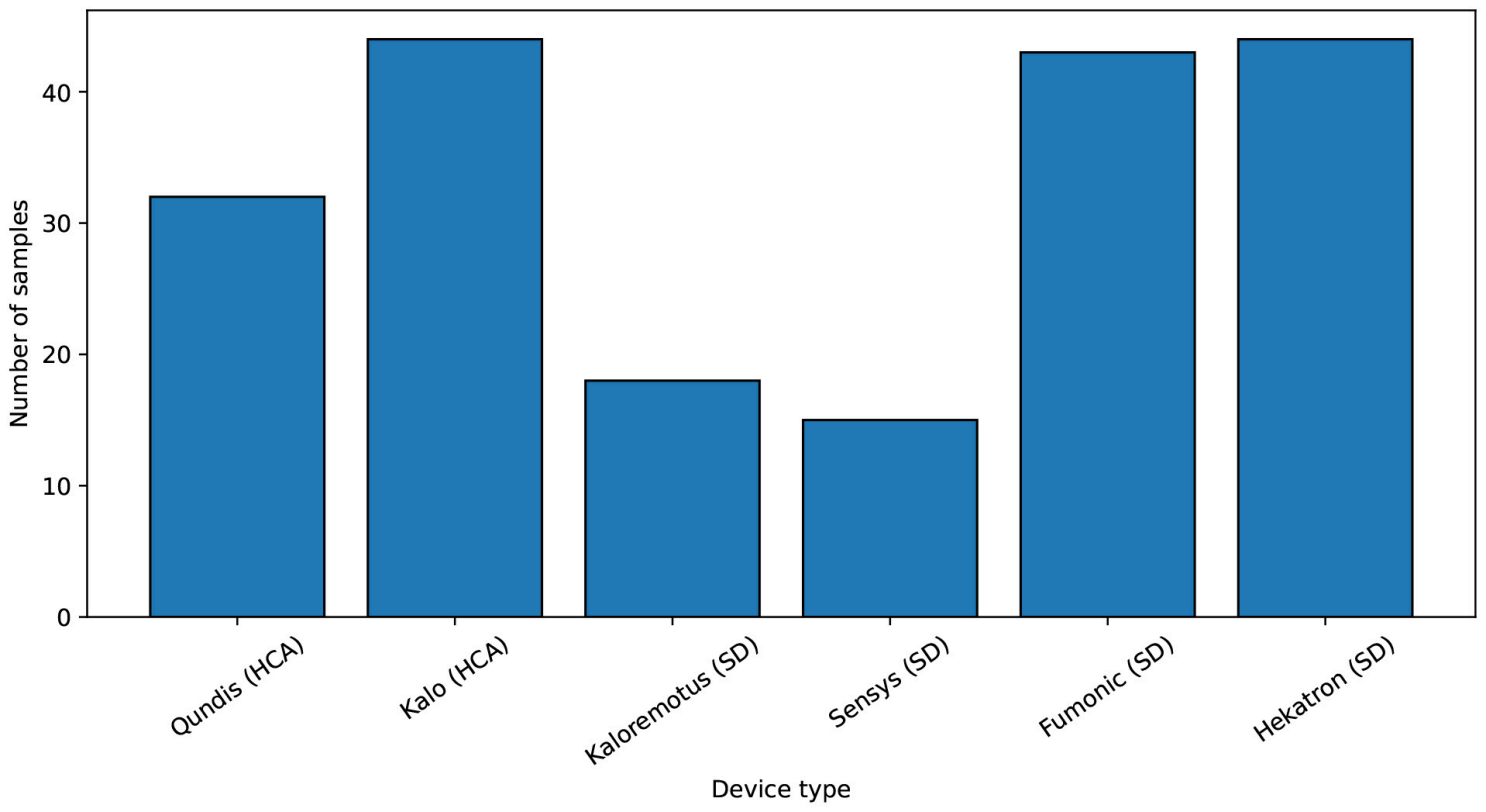
Distribution of images per device type.

For evaluation of our approaches, we designed a sequence-prediction dataset originating in the robotic recycling of waste electronic devices. The dataset consists of 196 images of 6 different electronic devices—4 types of smoke detectors and 2 types of heat-cost allocators. Figure 7 shows the distribution of images across device types. Here, SD denotes smoke detector and HCA denotes heat-cost allocator. Using this dataset, we addressed the following tasks: predicting the next disassembly action step, predicting the action which led to the current state, listing the remaining actions, determining whether the image depicts the final action, and predicting the general class of the object (e.g., smoke detector). Successfully predicting the general class of the object reduces the number of valid actions, since some disassembly actions are object-class specific, which is mentioned in the prompt provided to the VLM. The dataset contains sequences of images of a variety of discarded electronic devices, showing each device in different states of disassembly. For each image, ground-truth data regarding the device name and type, next disassembly action, the last applied disassembly action, the number of remaining disassembly actions, and the list of follow-up disassembly actions was provided. Additionally, the end-states of disassembly are labeled as such.

The current and next action step prediction encompasses eight different robotic actions. The list of possible actions includes:

- placing the object into a CNC machine,
- placing the object into a clamping vise,
- performing robotic levering on the object,
- cutting the object within a linear pneumatic cutter,
- performing a rectangular CNC cut,
- performing a circular CNC cut,
- CNC cutting of battery contacts, and
- removing a battery by wiggling.

To evaluate the RAG image query improvement and the performance of different embedding models, the initial dataset was used to create a bounding-box annotated dataset for cropping, where we labeled the electronic device along with the relevant neighboring machine tools (e.g., a clamping device), since this information provides the required context for making predictions.

### 3.2 Evaluation metrics

#### 3.2.1 Statistical significance testing

To evaluate whether the improvement between various methods is statistically significant, we apply McNemar's test (McNemar, 1947), which is appropriate for paired dichotomous outcomes (each model's prediction is correct or incorrect).

#### 3.2.2 Evaluation of RAG image query optimization

For both the grid-based and bounding-box-based methods, the following evaluation metrics are calculated after performing RAG query optimization:

- coverage *c*: the proportion of labeled polygon that was captured by VLM crop,
- image data removal *r*: the proportion of area that was removed from the initial image.

Here, coverage *c* is calculated as follows


$$\begin{array}{l} c=\frac{\text{Area}\left(P\cap G\right)}{\text{Area}\left(G\right)}, \end{array}$$ (4)

where *P* denotes the predicted bounding box, and *G* is the ground truth polygon. Image data removal *r* is calculated as


$$\begin{array}{l} r=1-\frac{\text{Area}\left(P\right)}{\text{Area}\left(\text{Ω}\right)}, \end{array}$$ (5)

where Area(Ω) denotes the area of the input image.

#### 3.2.3 Evaluation of image embedding model retrieval accuracy

The performance of a RAG system depends on the database quality—the similarity of database examples, as compared to the input queries—and the performance of the embedding models used. The key criteria are:

- Top-1 retrieval accuracy: The percentage of cases where the top-1 database-retrieved image matches the query image in device type and disassembly step, and
- Rank of correct result: The top-n number, where retrieved data point *n* matches the query image. This indicates how many RAG elements the VLM should process before finding a match in case of an inaccurate result from the image embedding model.

To quantify the effectiveness of RAG image query optimization on baseline RAG retrieval performance, we evaluated two aspects: (i) the performance of different open-source embedding models, and (ii) the effect of cropping on the performance of the embedding models, compared to vector retrieval using full-size, uncropped images.

To objectively compare the retrieval performance of different embedding models for the “Rank of correct result” criterion, we fitted exponential and log-normal distributions to the corresponding rank histograms and used the Akaike Information Criterion (AIC) to select the better-fitting distribution (Akaike, 1974). Based on this criterion, the log-normal distribution was selected and used to summarize and compare the retrieval-rank distributions across embedding models.

The following open-source embedding models are evaluated:

1. OpenAI CLIP small (*openai/clip-vit-base-patch32*) (Radford et al., 2021).
2. OpenAI CLIP large (*openai/clip-vit-large-patch14-336*) (Radford et al., 2021).
3. LAION CLIP large (*laion/CLIP-ViT-H-14-laion2B-s32B-b79K*) (Schuhmann et al., 2021).
4. SigLIP2 large (*google/siglip2-large-patch16-512*) (Tschannen et al., 2025).

### 3.3 Action prediction

Several types of models are evaluated, including classifiers based on convolutional neural networks (CNNs) and transformers, a dummy random classifier, as well as open source and commercial VLMs. For VLMs, the system prompt always included the list of available actions, including their preconditions and effects. The VLM model is given a list of possible valid outputs. All algorithms are only required to select an action, while the parameters for each action are predicted by specific custom subroutines. The evaluation metric for different tasks described in Section 3.1 is classification accuracy.

We evaluate a variety of image classification models, along with VLMs in different settings. The following image classification models are evaluated:

- CLIP (*openai/clip-vit-base-patch32*) (Radford et al., 2021),
- ResNet-152 (*microsoft/resnet-152*) (He et al., 2015), and
- VisionTransformer (*google/vit-base-patch16-224*) (Dosovitskiy et al., 2021).

Two open-source VLMs along with a single commercial VLM are evaluated:

- Qwen2.5-VL-72B-Instruct with full-precision weights (Yang et al., 2025a),
- Qwen3-VL-30B-A3B Mixture-of-Experts model with full precision weights (An et al., 2024), and
- OpenAI's GPT-4o (Hurst et al., 2024).

For zero-shot action prediction experiments, i.e. action prediction without using RAG, the models are provided with a query image showing the electronic device to be disassembled. The prompt includes:

- A general instruction to analyze the image in detail, focus on the environment (e.g., flat table, CNC machine, ...), the shape and color of the device, and any visible internal components.
- A detailed description of the available robotic workcell actions, including their preconditions and effects.
- A definition of all prediction tasks—previous step, next step, remaining steps, and whether the next step is the last step—together with the set of valid outputs for each task, and
- The previously executed robotic action.

In addition to the zero-shot action prediction, several RAG-based variants are evaluated. In these configurations, the zero-shot prompt is augmented with additional retrieved data:

- Baseline Single-RAG
  The prompt is extended with the most similar database example retrieved using the image embedding model. The retrieved entry consists of a candidate image and its associated ground-truth QA pairs.
- Bad-Single-RAG
  A deliberately incorrect database image is randomly selected – showing either a different electronic device or a different disassembly step. As in the Single-RAG, the image and its ground-truth text are appended to the VLM prompt. This setup evaluated how much action prediction accuracy degrades when a wrong database example is returned by RAG.
- VLM-guided RAG
  The retrieval process described in Section 2.4 is used, combining similarity information from the image embedding model with VLM reasoning over the knowledge tree. The highest-ranked image and its corresponding text are added to the VLM prompt, analogously to the other RAG settings. Additionally, detailed metadata from the retrieval process is recorded (e.g., image scores, VLM-generated justifications, the set of evaluated knowledge tree leaves, and the final selected image), enabling a separate, fine-grained analysis of the search procedure itself. This approach is denoted as **KG-Search** in the result figures.

For fine-tuned models, we use 5-fold cross-validation with splits performed at the device-folder level. The dataset consists of six device folders, and all images from a given device folder are assigned to the same subset. In each fold, the data are split into training, validation, and test sets using an approximately 70–10–20 ratio. This prevents images from the same device from appearing in multiple subsets.

For models that are not fine-tuned, evaluation is performed on the full dataset, and the corresponding results on the figures are reported under the label “combined.”

For RAG-based approaches, the retrieval database contains two representative images for each disassembly step of each device. The images were manually selected to clearly show the relevant device state, while avoiding redundant views. Specifically, for each of the *n* devices and each of their *m* disassembly steps, two representative images are included instead of all available images, which typically consist of approximately seven images per step. During evaluation, the query image is removed from the retrieval database to prevent trivial self-matches. Since the database contains two images per disassembly step, at least one valid reference image remains available for retrieval.

## 4 Results

This section presents a comprehensive evaluation of the different parts of our VLM-guided RAG framework. The effectiveness of different VLMs in the image query improvement step is quantified in Section 2.2, showing that even VLMs not specifically trained for object detection are effective in removing irrelevant image parts.

An evaluation of different open source image embedding models and their retrieval accuracy on both original and improved (cropped) images is shown in Section 4.2. Our analysis shows that image cropping results in a significant increase of retrieval accuracy. The retrieval accuracy using our VLM-guided search and re-ranking compared to baseline image embedding models is shown in Section 4.3, demonstrating a statistically significant improvement.

Zero-shot action prediction results for image classification models are shown in Section 4.4.1. In Section 4.4.2, we show that fine tuning of VLMs is not effective on our small dataset. However, due to computational budget constraints, we could only fine-tune the 8B-parameter *Qwen3* variant, which is relatively small compared to the 72B-scale VLMs that we use for zero-shot inference experiments.

The action prediction accuracies for zero-shot, Single RAG and VLM-guided RAG approaches are shown in Section 4.4.3. Since prediction latency is an important factor in real-world usability, a comparison of prediction latencies of zero-shot approaches and our VLM-guided RAG is provided in Section 4.5. Finally, an analysis of failure modes for various models and methods is discussed in Section 4.6.

### 4.1 RAG image query optimization

The results obtained with grid and bounding-box cropping are shown in Table 1. The iterative bounding box cropping method demonstrates superior results compared to grid-based cropping, which we suspect is due to it enabling higher resolution of selecting relevant image parts. The *Qwen3-VL-30B* model demonstrates the best performance, removing **49.6%** of the image while retaining **95.4%** coverage of key image regions.

**Table 1:** Coverage and image data removal metrics for VLM crops.

Model	Cov. (%)	Data removal (%)
Qwen2.5-VL-72B - grid cropping	**95.85**	7.3
Qwen3-VL-30B - grid cropping	73	**44.2**
GPT-4o - grid cropping	78.5	38.4
Qwen2.5-VL-72B - bbox cropping	92.8	39.7
Qwen3-VL-30B - bbox cropping	**95.4**	49.6
GPT-4o - bbox cropping	81.5	**71.5**

Bold values indicate the best-performing result in each column, evaluated separately for the grid-based and bounding-box-based cropping methods.

To ensure fair comparison between different models that do not execute the image query optimization step (i.e., image classification models), all images are manually cropped for further sequence prediction tasks.

### 4.2 Retrieval accuracy of image embedding models

**Figure 8:**
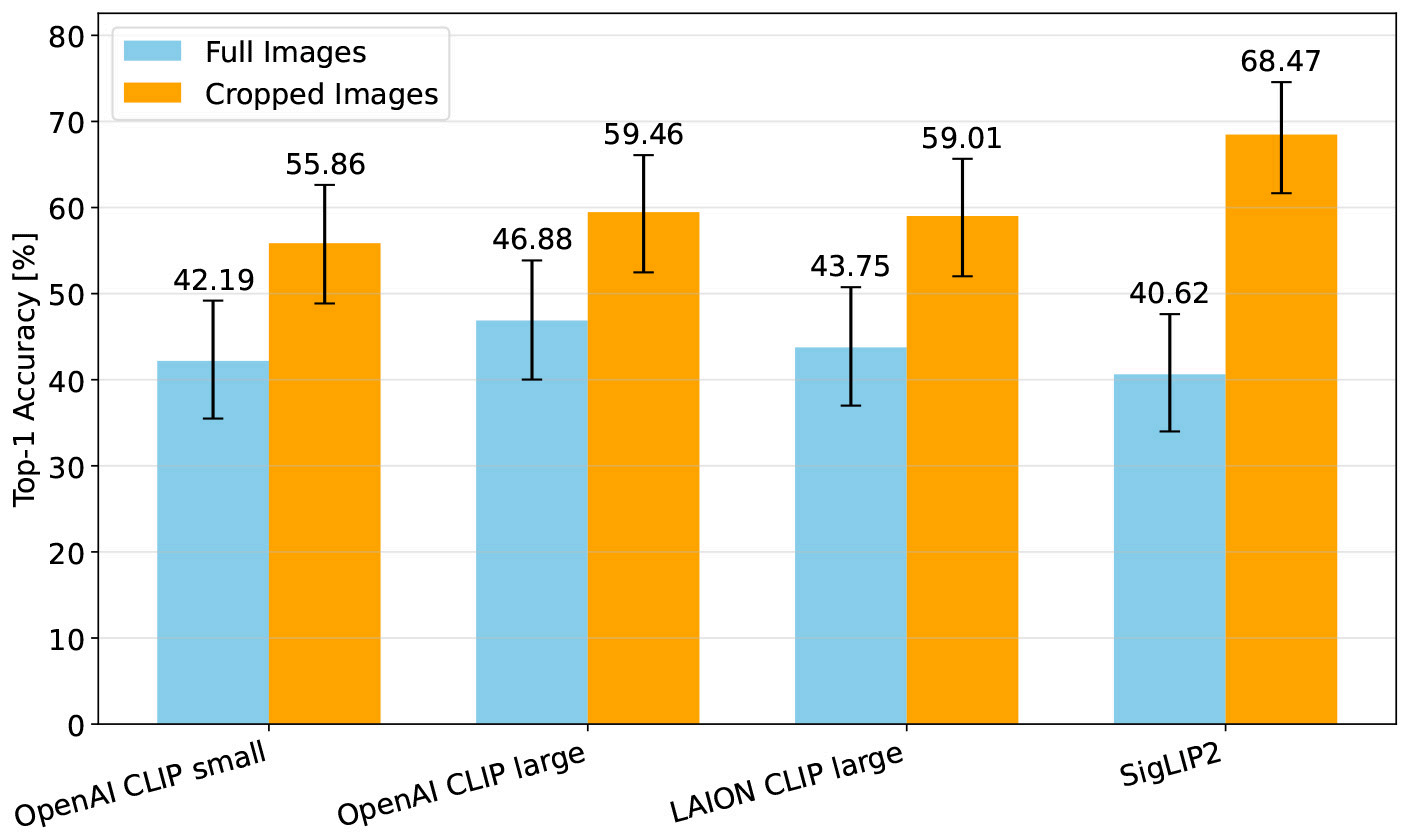
Top-1 retrieval accuracy of evaluated models, with 95% Wilson binomial confidence intervals.

Top-1 retrieval accuracy of the evaluated embedding models on both cropped images and full-size images is shown in Figure 8. Error bars indicate 95% Wilson binomial confidence intervals, computed using *n* = 196. In all cases, cropping of the images improves the Top-1 retrieval performance of evaluated embedding models. Based on the absolute accuracies shown in Figure 8, the corresponding relative improvements range from **32.468%** for *OpenAI CLIP small* to **68.5%** for *SigLIP2*.

On full, non-cropped images, *OpenAI CLIP Large* shows the highest Top-1 accuracy of **46.88%**. On cropped images, the *SigLIP2* model displays the highest Top-1 accuracy of **68.47%**, representing a relative improvement of **22.6%** over the smaller base *OpenAI CLIP* model. This is due to larger model size, the larger size of the training dataset, and enhancements in the model architecture (Tschannen et al., 2025).

In addition to Top-1 accuracy, we evaluate the rank of the correct result for each model. While Top-1 accuracy measures whether the correct item is retrieved as the first result, it does not capture how close the model is in cases where the top prediction is incorrect. The rank metric addresses this limitation by indicating how deep in the retrieval list the correct result appears.

**Figure 9:**
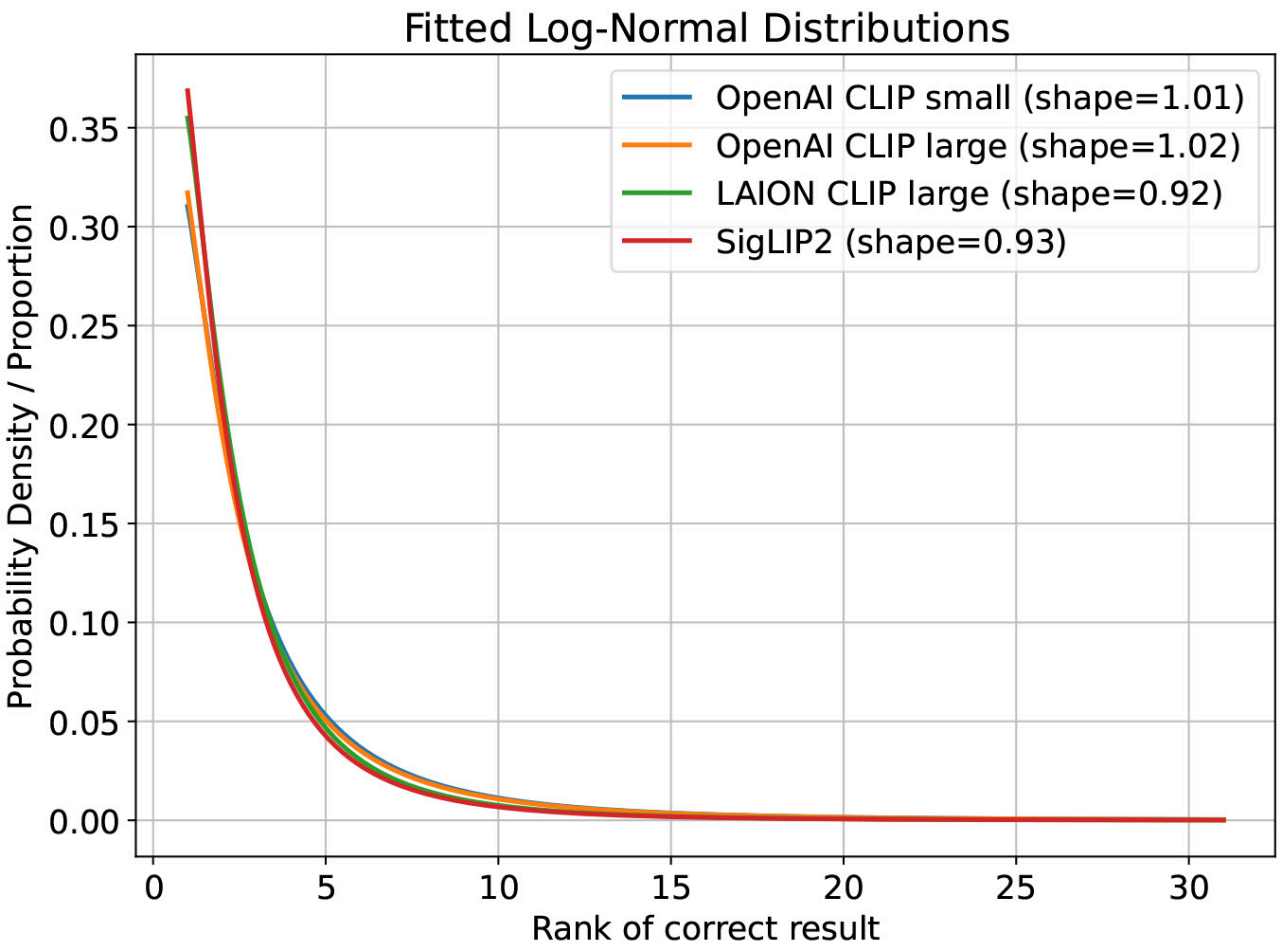
Results of fitting the log-norm distribution on rank of correct result histogram.

To quantitatively analyze the distributions of the rank of the correct result for various models, we fit the log-normal distribution to the empirical data. The resulting fitted distributions are shown in Figure 9.

Among the evaluated models, *SigLIP2* achieves the best performance in terms of Top-1 retrieval accuracy, while only slightly lagging behind *LAION CLIP* in ranking quality, with a log-normal shape parameter σ = 0.93 compared to 0.92 for *LAION CLIP*. For the *SigLIP2* model, the mean rank of the correct result is **2.43**, which indicates that, on average, the correct match is found within the top-3 retrieved results.

These results are particularly relevant for RAG, where multiple top-ranked items are typically used. Therefore, *SigLIP2* is selected as the embedding model for subsequent experiments.

### 4.3 Retrieval accuracy of VLM-guided RAG

Before testing action prediction accuracy, we separately evaluated the retrieval performance of our VLM-guided re-ranking and search expansion algorithm compared to baseline RAG using the *SigLIP2* embedding model. The resulting absolute and relative improvements for each model are shown in Table 2.

**Table 2:** Retrieval accuracy comparison across models.

Model	Accuracy (%)	Abs. Impr. (%)	Rel. Impr. (%)
Base RAG	68.47	–	–
Qwen2.5-VL-72B	84.57	16.1	23.5
Qwen3-VL-30B	85.41	16.94	24.7
GPT-4o	**89.29**	**20.82**	**30.4**

Bold values indicate the best performance in each column.

The *GPT-4o* was the most accurate, at the cost of longest prediction latency due to larger model size. We observe that the *Qwen3-VL-30B* model provides the best tradeoff between reasonable search times and accuracy. This is due to its mixture-of-experts architecture (Artetxe et al., 2022), which during inference activates only a smaller subset of parameters.

To evaluate whether the differences in retrieval performance between different models using our VLM-guided re-ranking and search expansion algorithm are statistically significant, we performed McNemar's test, as described in Section 3.2.1. The results are shown in Table 3. It can be seen that the performance difference between *Qwen2.5-VL-72B* and *Qwen3-VL-30B* is not statistically distinguishable (*p* = 1.0), while the differences between *GPT-4o* and both *Qwen2.5-VL-72B* and *Qwen3-VL-30B* are statistically significant at the 5% level (*p* = 0.0158 and *p* = 0.0268, respectively), indicating that *GPT-4o* model achieves consistently higher retrieval accuracy in the VLM-guided re-ranking and search expansion setting.

**Table 3:** McNemar's test results comparing VLM-guided re-ranking and search expansion retrieval accuracy.

Models	GPT-4o	Qwen2.5	Qwen3
**GPT-4o**	/	0.0158	0.0268
**Qwen2.5**	/	/	1.0

### 4.4 Action prediction and ablation study

We performed an ablation study to test the effectiveness of the VLM-guided RAG approach for next action prediction using both open-source and commercial VLMs. Firstly, we compared results obtained with VLM-guided RAG to the results obtained by fine tuning image classification models. Secondly, VLM-guided RAG approach was compared to fine tuning a small VLM. Finally, VLM-guided RAG was compared to accuracy of zero-shot prediction with VLMs.

#### 4.4.1 Image classification models

**Figure 10:**
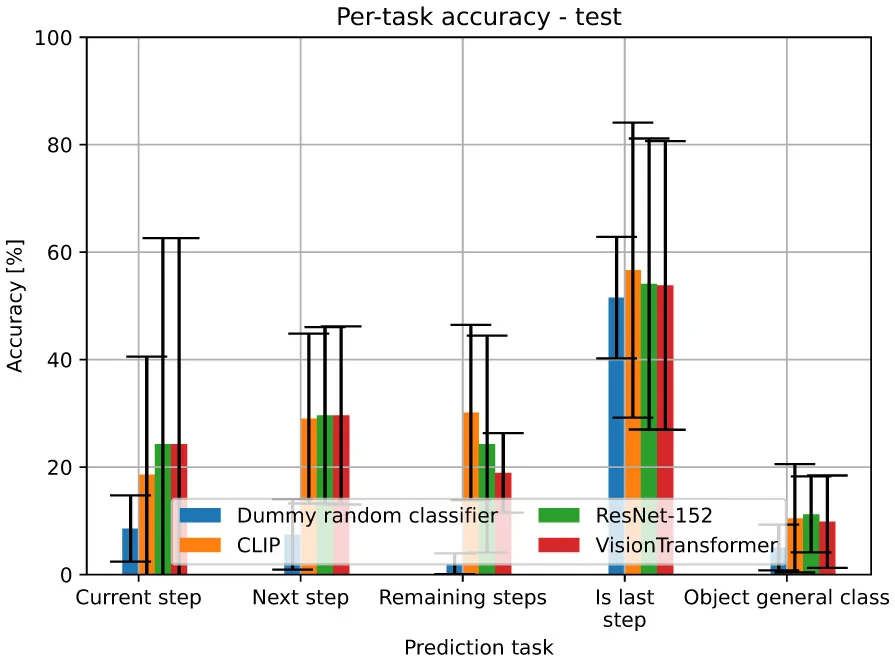
Results of fine-tuning vision models.

For single task prediction, we fine tuned several classifiers based on CNNs and transformer-based models, as described in Section 3.3. The algorithms are also compared to random choice, as suggested by Metz et al. (2015). Models were trained and evaluated on each task separately. A maximum of 30 epochs were used for training. Early stopping was implemented based on validation loss, where training was stopped when validation loss did not improve after 2 epochs. The models with the lowest validation loss were evaluated. The results are shown in in Figure 10. They show that although the performance on the training set is high, the models can not generalize due to small dataset size, as well as lack of relevant textual input. The trained models outperform the random classifier.

#### 4.4.2 VLM with fine tuning

**Figure 11:**
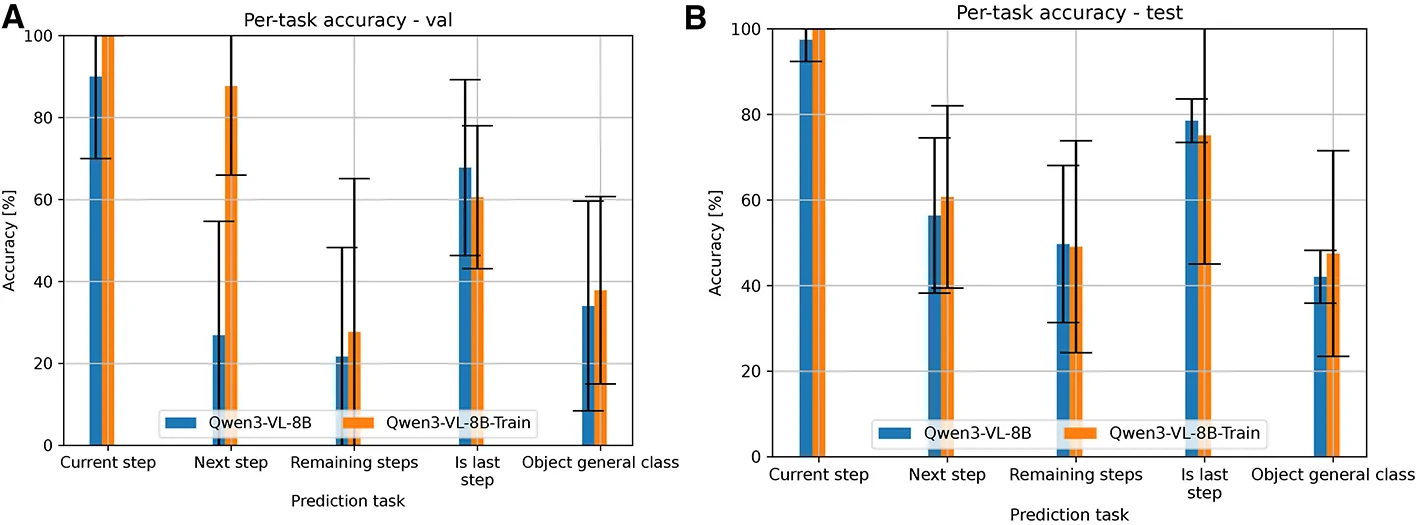
Comparison of accuracy between pre-trained and fine-tuned Qwen3-VL-8B. **(A)** Validation set. **(B)** Test set.

The results of fine tuning a VLM are shown in Figure 11. In both cases, results are compared to the baseline, non-fine-tuned model. The VLM was trained up to a maximum of 20 epochs, however early stopping based on validation loss was used to prevent overfitting (Prechelt, 2000). In most cases, the training was finished by epoch 3.

The results show that while performance improves on the validation set, the accuracy gains on the test set are minimal. This is likely due to the small dataset used, which limits generalization. A larger and more diverse dataset would likely improve the effectiveness of fine-tuning. Additionally, due to computational limitations, fine-tuning cannot be performed on larger models, therefore a smaller model with 8B parameters was used, which in itself decreases action prediction accuracy.

#### 4.4.3 VLMs without fine tuning

**Figure 12:**
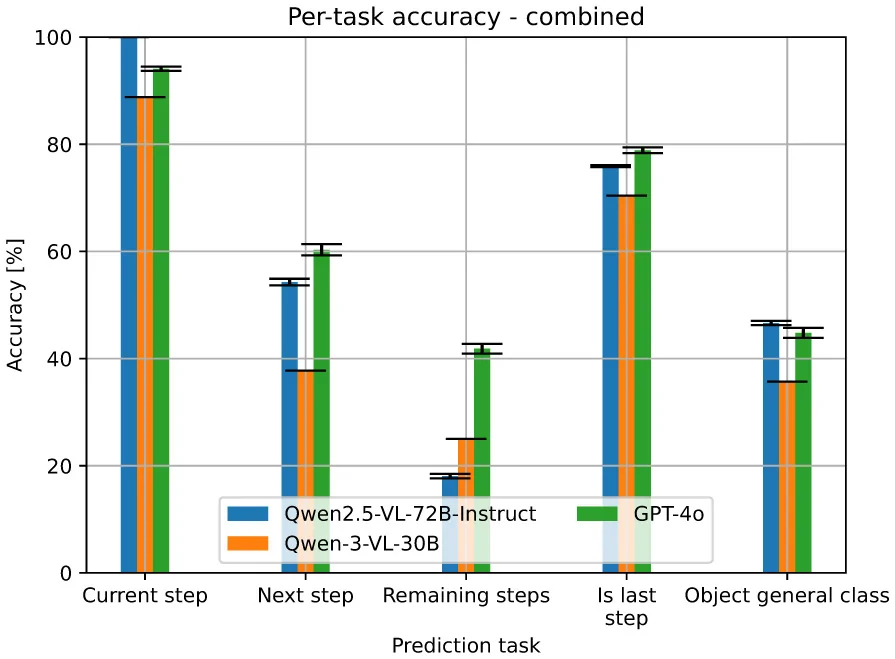
Zero-shot accuracies of various models.

In Figure 12, the action prediction accuracy results using pre-trained VLMs for each task are shown. Compared to Figure 10, it is evident that VLMs outperform image classification models like ResNet, CLIP, and others. Image classification models can not be used effectively, primarily due to the small training dataset size. The higher accuracy of even pre-trained VLMs can also be attributed to richer information provided by the relevant text describing action possibilities, and the prior experience VLMs gain by pre-training on large datasets with general world knowledge. To ensure that VLM responses are consistent and deterministic, the model selects the most likely output at each step of the prediction process, a method known as greedy decoding.

**Figure 13:**
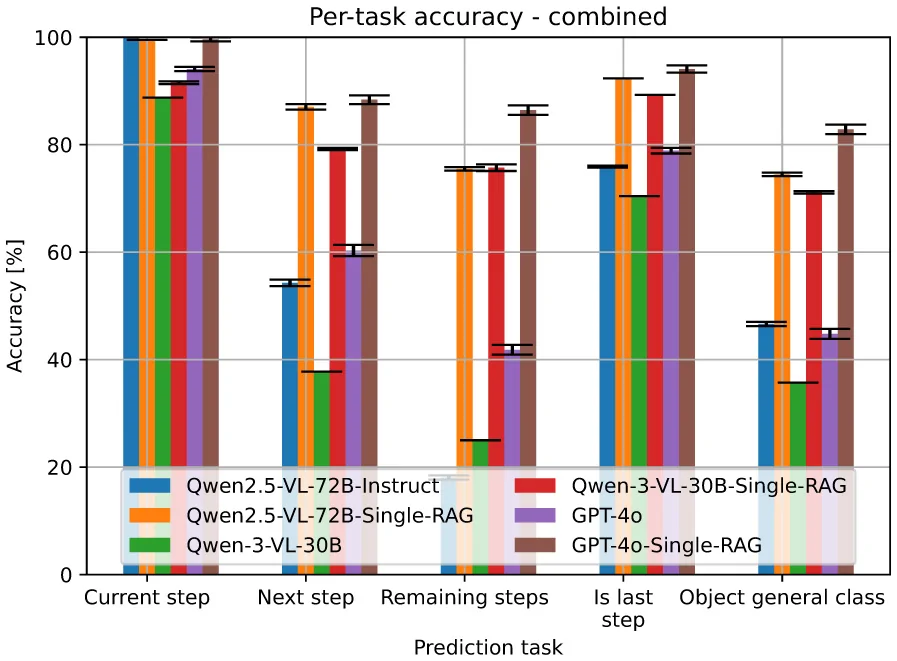
Results of zero-shot vs. Single-RAG.

For the zero-shot predictions, shown in Figure 13, there is a clear difference in accuracy between the different models. The commercial *GPT-4o* model outperforms both Qwen variants, reaching 61% accuracy in next-step prediction. However, such accuracy is insufficient for industrial use. The prediction of the “current step” (which led to the currently-visible image) is meant to separately evaluate the performance of the Language Model (LM) part of the VLM, since the required information is directly included in the prompt and needs to be extracted from the context by the VLM. Notably, it can be seen that the *Qwen3-VL-30B* fails more frequently on this task than the other evaluated models, indicating reduced instruction-following reliability in this specific setting. This is possibly due to the smaller number of parameters in the *Qwen3-VL-30B* model compared to other models.

Compared to the zero-shot accuracy, the baseline Single-RAG approach exhibits significantly higher prediction accuracies, as shown in Figure 13, at minimal inference latency increase (see Section 4.5). Note that the accuracy could be further improved by increasing the number of RAG examples in the dataset.

**Figure 14:**
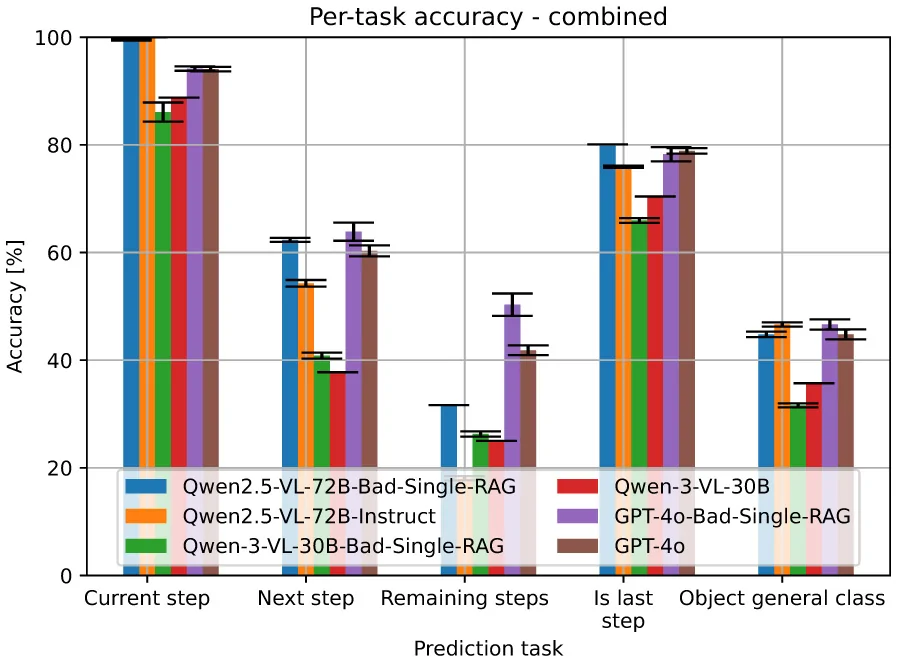
Results of Bad-Single-RAG vs zero-shot.

In the case of Bad-Single-RAG, where a VLM is deliberately given a wrong candidate image and its corresponding text, it can be observed that in most cases, the VLMs are able to recognize that the candidate image is irrelevant and also that the provided corresponding text is not helpful, as shown by results in Figure 14. In some cases, the accuracy is even higher than the zero-shot baseline. We hypothesize that the additional example helps “ground” the VLM in seeing which action is **not** applicable, thereby reducing the number of applicable actions.

To evaluate whether Bad-Single-RAG results are statistically distinguishable from the zero-shot variation, we perform McNemar's test. The results are shown in Table 4. At the 5% significance threshold, the difference is statistically insignificant.

**Table 4:** McNemar's test results comparing baseline zero-shot predictions to Bad Single RAG.

Model family	Compared variants	χ^2^(1)	*p*-value
Qwen2.5-VL	Zero-shot vs. Bad Single RAG	3.67	0.055
Qwen3-VL	Zero-shot vs. Bad Single RAG	0.129	0.72
GPT-4o	Zero-shot vs. Bad Single RAG	0.114	0.735

**Figure 15:**
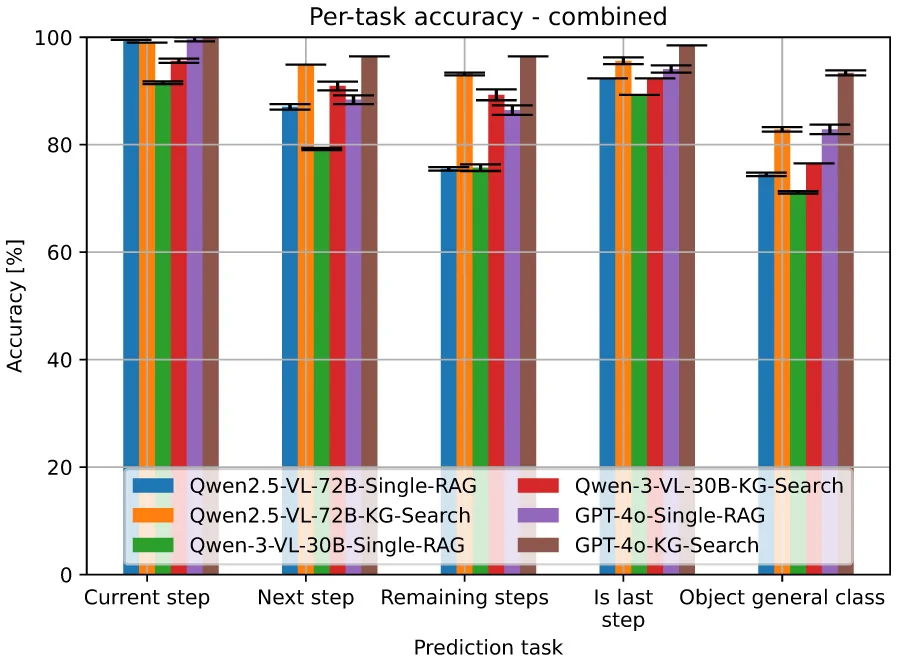
Results of Single-RAG vs. VLM-guided RAG.

Compared to the Single-RAG approach, it can be seen that in all cases, VLM-guided RAG approaches (abbreviated as **KG-Search** in the figures) exhibit increased accuracy, as shown in Figure 15. The difference between Single-RAG and VLM-guided RAG is shown to be statistically significant at the 5% threshold for all models, as shown in Table 5.

**Table 5:** McNemar's test results comparing Single-RAG to VLM-guided RAG.

Model family	Compared variants	χ^2^(1)	*p*-value
Qwen2.5-VL	Single-RAG vs. KG-Search	8.45	0.0037
Qwen3-VL	Single-RAG vs. KG-Search	9.03	0.0027
GPT-4o	Single-RAG vs. KG-Search	8.45	0.0037

**Figure 16:**
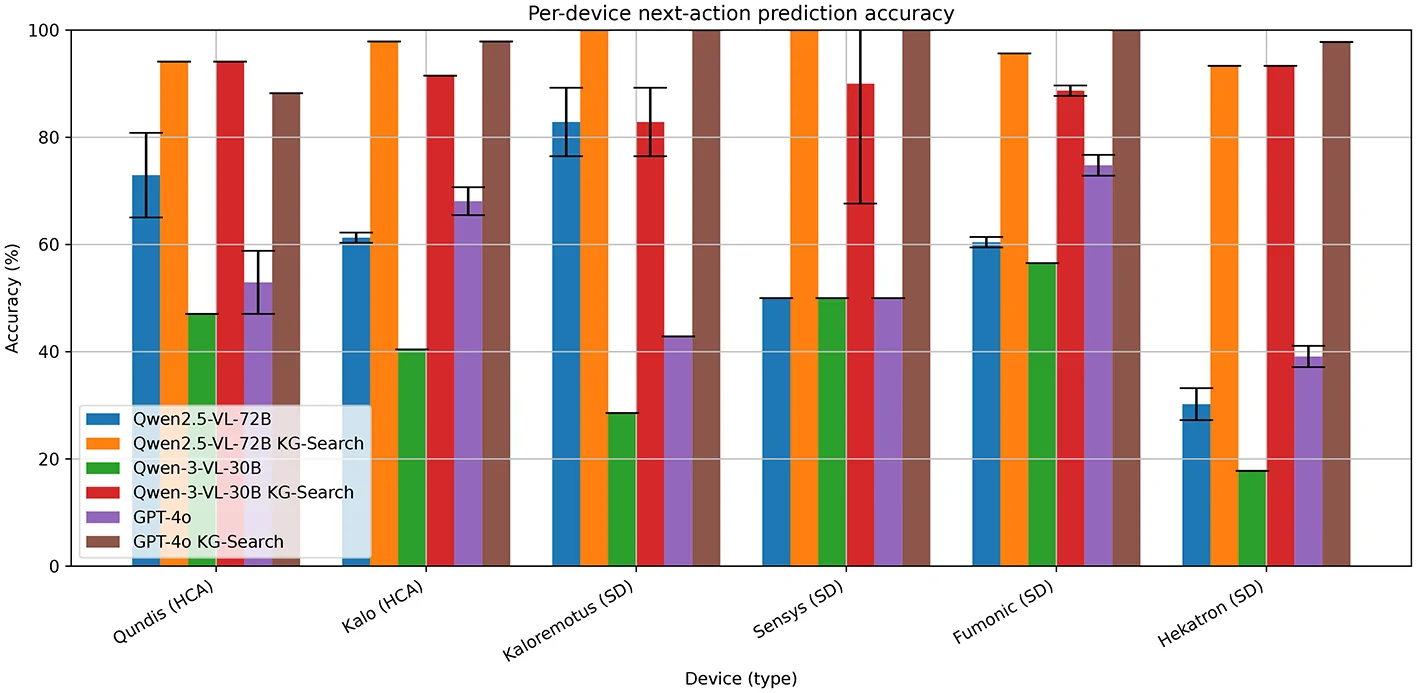
Accuracy comparison between the zero-shot VLM baseline and the VLM-guided RAG method across device types.

Lastly, Figure 16 compares the accuracy of the baseline zero-shot VLM next-action prediction and the VLM-guided RAG method across different device types. The zero-shot baseline exhibits substantial variation in accuracy depending on the device type, indicating that its performance is sensitive to the visual characteristics of the target device. In contrast, the VLM-guided RAG method achieves consistently higher and more uniform accuracy across device types, suggesting improved robustness to device-specific differences.

### 4.5 Prediction latency

Prediction latency is a key criteria of system usability in real-world applications, since industrial robots are expected to operate with minimal downtime. Nevertheless, in disassembly of waste electronics, action prediction accuracy is more important than prediction latency, since incorrect predictions may lead to severe consequences, e.g. battery fires may occur if incorrect CNC cutting methodology is utilized.

In Table 6, the mean prediction latencies and standard deviations are presented for various algorithms and cases. All results are presented for the Qwen3-VL 30B model, since it performs best in VLM-guided RAG, as well as being competitive in the zero-shot scenario. While the mean prediction latency for one image (zero-shot prediction) is **5.21 seconds**, prediction latencies for RAG-augmented cases, which require processing two images and additional image text, takes on average **6.15 s**. Prediction latency in case of VLM-guided RAG is longer, since in the best case scenario, at least 2 extra images must be processed, i.e. the VLM must additionally confirm that the retrieved image matches the query. In this case, the mean prediction latency increases to **9.41 s**. Performing a full knowledge tree search is significantly more expensive, with a mean elapsed time of **157.25 s**. However, due to there being many cases where the first retrieved image is judged correct, VLM-guided RAG search takes on average **37.71 s**. Importantly, the median latency of the overall KG-search procedure is only **8.95 s**, indicating that in most cases the prediction time is substantially lower than the mean. The larger mean and standard deviation are mainly caused by the smaller subset of cases in which the Top-1 result is incorrect and the full knowledge-tree search is required. In practical deployment, the RAG database can also be improved online by the operator. If certain device types or visual configurations repeatedly require the full knowledge-tree search, the corresponding validated images and action labels can be added to the database. This increases database coverage and can improve the likelihood of correct Top-1 retrievals for future queries, thereby reducing the frequency of expensive knowledge-tree searches over time.

**Table 6:** Prediction times for different methods.

Model	Prediction time mean [s]	Pred. t. std [s]	Median [s]
Zero-shot (single image)	**5.21**	**0.81**	**5.10**
Single RAG (two images)	6.15	0.89	6.02
KG search—Top-1 result correct	9.41	2.56	8.87
KG search—Top-1 result incorrect	157.25	86.10	108.80
KG search—overall	37.71	70.52	8.95

Bold values indicate the best performance in each column.

### 4.6 Failure modes

We perform an analysis of failure modes to determine the reasons for incorrect action prediction results. Three separate cases are considered: (1) the cases in which Single-RAG retrieval fails, (2) the cases in which VLM-guided RAG retrieval fails, and (3) the cases in which next-action prediction fails.

**Figure 17:**
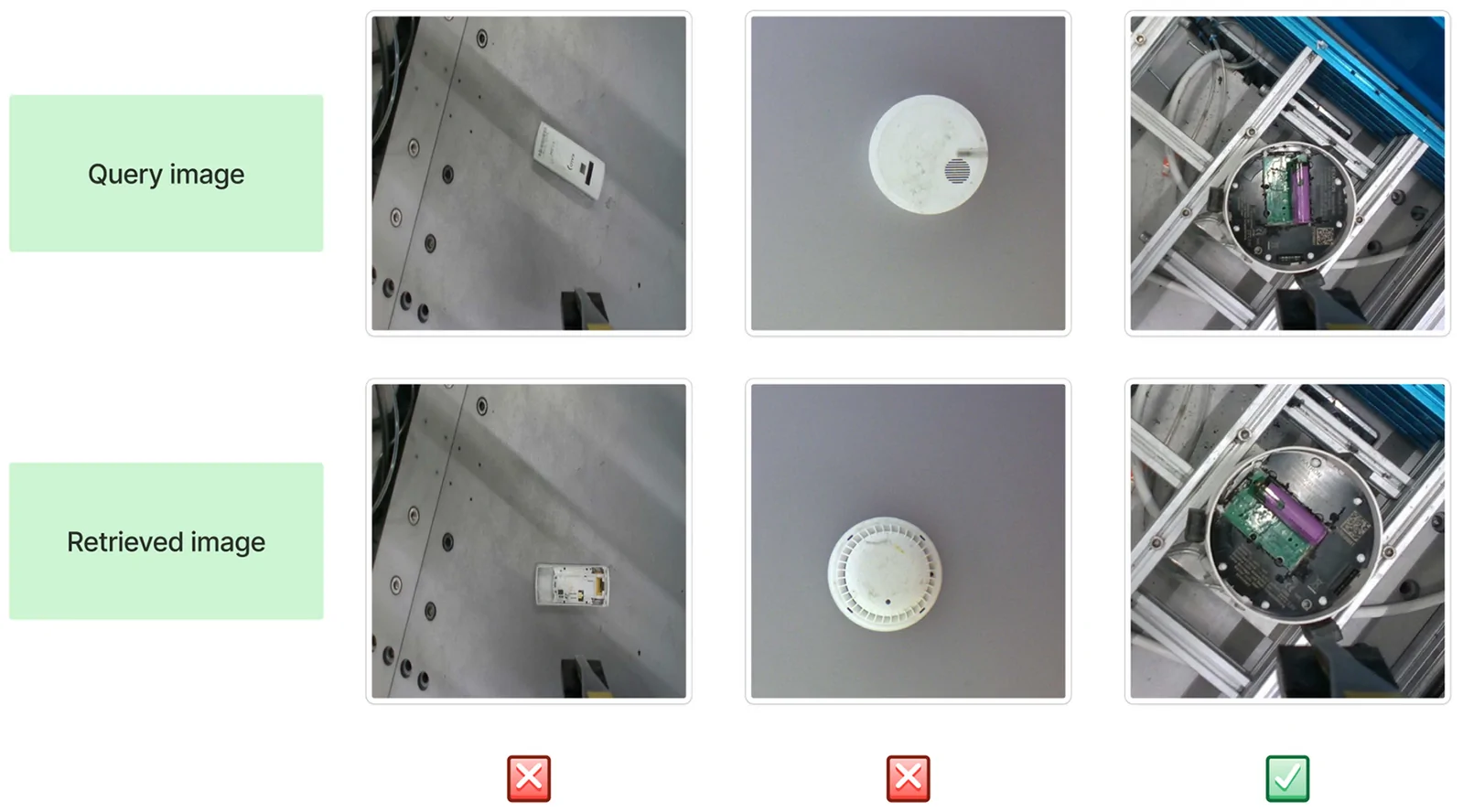
Example failure cases for Single RAG image retrieval.

For the failure cases of Single-RAG retrieval, shown in Figure 17, we observe that retrieval fails when images before and after the disassembly operation are almost the same, e.g., before and after battery contact cutting is performed within the CNC mill. This is due to the RAG retrieval step only determining similarity between images, but not using additional textual information, e.g., the previously performed step, which can additionally help to delineate and improve retrieval in the case of very similar images. Some example failure cases are shown in Figure 17.

**Figure 18:**
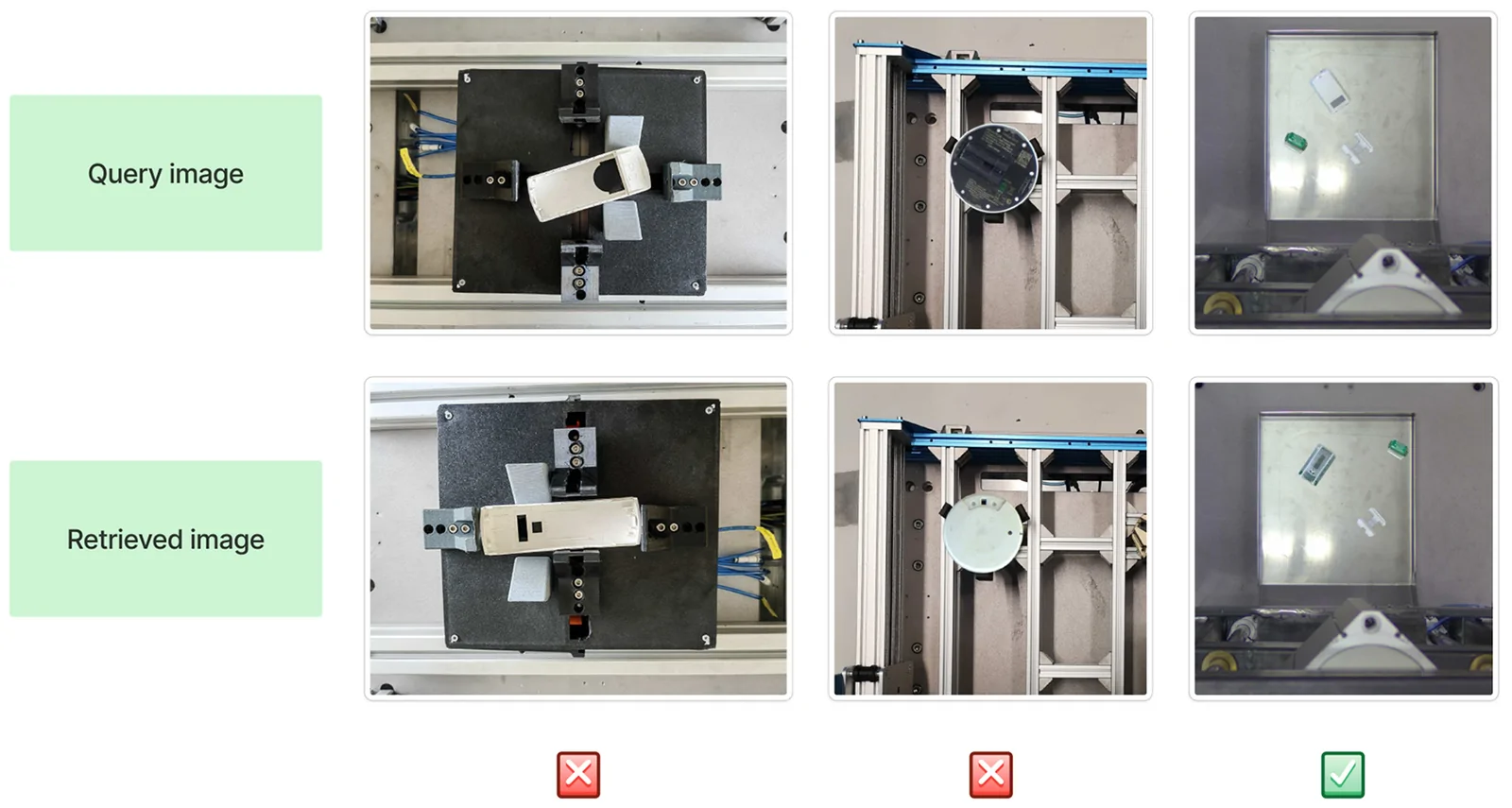
Failure cases for VLM-guided RAG.

In the case of VLM-guided RAG, out of 196 images, we observed only one total failure where the object in the query and retrieved image is clearly different, shown in the center of Figure 18. In other cases, the device types may be different, however, the images are semantically similar with same disassembly steps, which is why the next-action prediction accuracy is higher than would be suggested by the accuracy of the VLM-guided image retrieval. Additionally, most failure cases occur when the VLM confirms the most-similar RAG image as correct, thereby skipping the knowledge tree search phase. Accuracy would be higher in case of always forcing the knowledge tree search, but at the cost of significantly higher prediction times.

When evaluating the next action prediction errors, it can be seen that VLMs sometimes confuse circular and rectangular CNC cutting approaches, seemingly missing the extruded rectangular battery outline that may be present in smoke detectors. Since such a feature is easily distinguishable on depth images, future work could be aimed at enabling VLMs to use also depth information for better spatial comprehension.

### 4.7 Application in robotic workcell

The developed VLM-guided RAG approach was implemented in a modular robotic workcell (Simonic et al., 2021; Ude et al., 2025) as a Robot Operating System (ROS) node. Based on the device to be disassembled, the VLM-guided RAG module is utilized to retrieve a relevant data point and corresponding text from the database, based on the current snapshot of the workcell. The corresponding text contains the disassembly steps and the device type. This is then passed to the action prediction VLM, along with information about the available robotic disassembly skills, as shown in Figure 3. The additional information from the VLM-guided RAG retrieved data point improves the action prediction performance of the VLM, as shown in Figure 12. Our approach supports adaptation to the tested device families without task-specific fine-tuning, suggesting a practical route toward low-data deployment in industrial workcells. Adaptation is achieved by adding the required device-specific information, such as novel device images and disassembly steps, to the RAG database. Based on the selected action, custom sub-routines are then used to determine the corresponding action parameters, for example for CNC cutting.

## 5 Discussion and conclusions

We have presented an approach for robot action prediction utilizing a VLM along with a local database and RAG, where the VLM firstly improves the RAG input query image, then iteratively queries the database and determines the most suitable relevant images and text content. The retrieved best image and summarized text are used by the VLM in the context of next action prediction in a robotic workcell. We compared our VLM-guided RAG approach to a baseline approach not utilizing RAG (zero-shot), an approach utilizing a single-best retrieved RAG image (Single-RAG), the VLM-guided RAG, and an approach where the model is deliberately given a misleading image and corresponding text (Bad-Single-RAG).

Firstly, the image query improvement step was shown to be successful in improving the RAG database and the query image by removing unnecessary elements, while retaining the relevant electronic devices within the image crop. Secondly, a variety of open-source image embedding models were evaluated. It was shown that the image query improvement step significantly improves the retrieval performance of the baseline RAG system. Additionally, larger embedding models exhibit both higher top-1 and top-n retrieval accuracies. Thirdly, the zero-shot action prediction performance of both open-source and commercial models was evaluated. It was compared to the Bad-Single-RAG approach, where the models were deliberately given a misleading image, to simulate the case where the RAG database contains no relevant datapoints. It was shown that in this case, the performance of the models does not degrade. The Single-RAG approach was shown to significantly increase the action prediction accuracy compared to zero-shot predictions. Crucially, it led to a minimal prediction latency increase. Fine-tuning was not effective on our dataset due to its small size and was outperformed by RAG-based approaches. Finally, the VLM-guided re-ranking and search approach was evaluated, showing an increases both in the retrieval accuracy and the final action prediction accuracy. This approach was compared to Single-RAG and showed a statistically significant increase in action prediction accuracy for all models at the cost of extending the prediction latency, since the time required to perform the VLM-guided RAG step is not deterministic.

In conclusion, the proposed VLM-guided RAG approach improves robot action prediction compared with zero-shot VLM inference, a baseline RAG strategy, and the fine-tuned models evaluated in this work, achieving a statistically significant increase in prediction accuracy. By combining image query optimization, VLM-guided re-ranking, and search expansion, the method improves the performance of the robotic workcell's action-selection module and supports faster adaptation to the disassembly of new device types without requiring model fine-tuning, making it well suited to low-data robotic disassembly settings where collecting large labeled datasets is impractical.

## Data Availability

The datasets presented in this study can be found in online repositories. The names of the repository/repositories and accession number(s) can be found below: https://github.com/bkuster0/knowledge_tree.
