# Supplementary material for: VLM-guided retrieval augmented generation (RAG) for robot action prediction
Source: Front Neurorobot. 2026 Aug 25;20:1894674. doi: 10.3389/fnbot.2026.1894674 (PMC13550167; doi:10.3389/fnbot.2026.1894674)
Supplement: Supplementary file 1 [file Data_Sheet_1.zip › fig/embedding_model_eval/embedding_acc_combined_old.pdf]

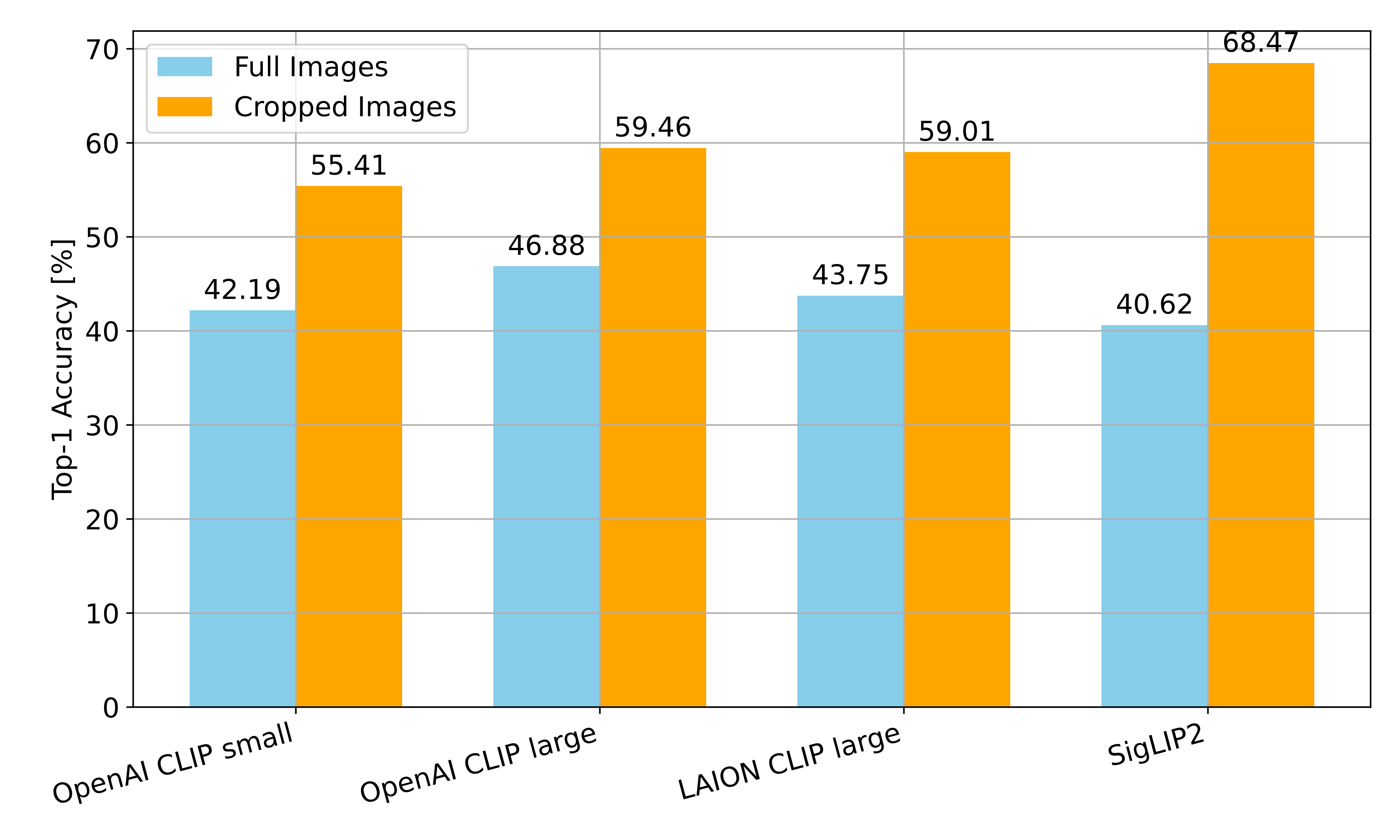

Full Images
Cropped Images
Top-1 Accuracy [%]
0
10
20
30
40
50
60
70
42.19
55.41
46.88
59.46
43.75
59.01
40.62
68.47
OpenAI CLIP small
OpenAI CLIP large
LAION CLIP large
SigLIP2
