# Supplementary material for: VLM-guided retrieval augmented generation (RAG) for robot action prediction
Source: Front Neurorobot. 2026 Aug 25;20:1894674. doi: 10.3389/fnbot.2026.1894674 (PMC13550167; doi:10.3389/fnbot.2026.1894674)
Supplement: Supplementary file 1 [file Data_Sheet_1.zip › fig/rag_process_v4.pdf]

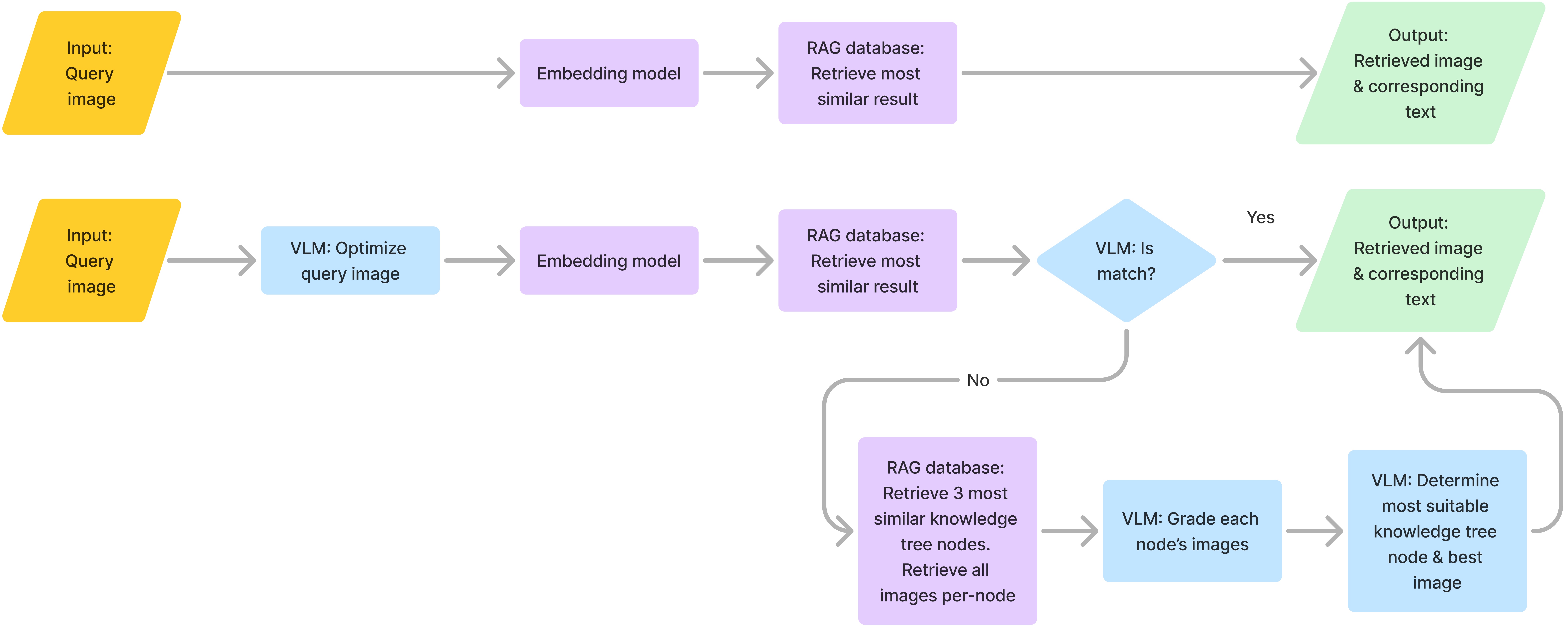
Input:
Query
image
Embedding model
RAG database:
Retrieve most
similar result
Output:
Retrieved image
& corresponding
text
Input:
Query
image
VLM: Optimize
query image
Embedding model
RAG database:
Retrieve most
similar result
VLM: Is
match?
Yes
Output:
Retrieved image
& corresponding
text
No
RAG database:
Retrieve 3 most
similar knowledge
tree nodes.
Retrieve all
images per-node
VLM: Grade each
node's images
VLM: Determine
most suitable
knowledge tree
node & best
image
