# Supplementary material for: VLM-guided retrieval augmented generation (RAG) for robot action prediction
Source: Front Neurorobot. 2026 Aug 25;20:1894674. doi: 10.3389/fnbot.2026.1894674 (PMC13550167; doi:10.3389/fnbot.2026.1894674)
Supplement: Supplementary file 1 [file Data_Sheet_1.zip › fig/action_prediction_v3.pdf]

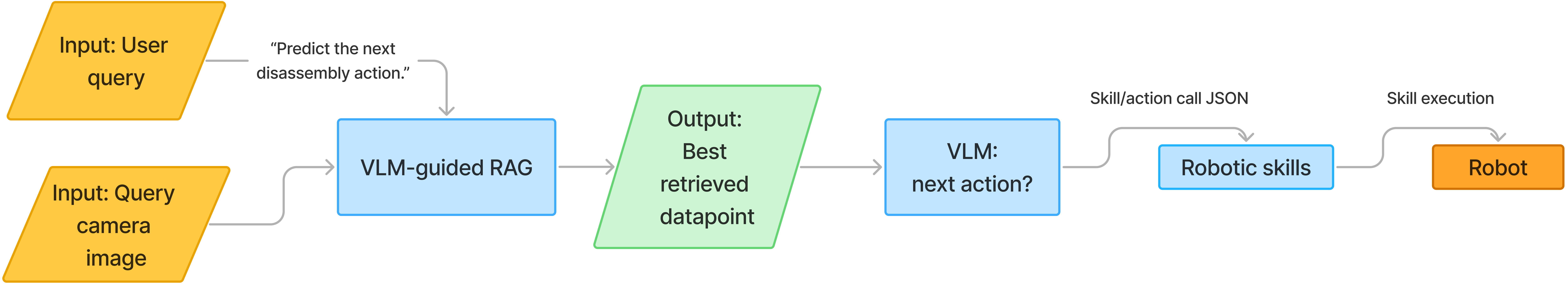
Input: User query
"Predict the next disassembly action."
Input: Query camera image
VLM-guided RAG
Output: Best retrieved datapoint
VLM: next action?
Skill/action call JSON
Robotic skills
Skill execution
Robot
