# Supplementary material for: VLM-guided retrieval augmented generation (RAG) for robot action prediction
Source: Front Neurorobot. 2026 Aug 25;20:1894674. doi: 10.3389/fnbot.2026.1894674 (PMC13550167; doi:10.3389/fnbot.2026.1894674)
Supplement: Supplementary file 1 [file Data_Sheet_1.zip › fig/finetune/multitask_accuracy_test.pdf]

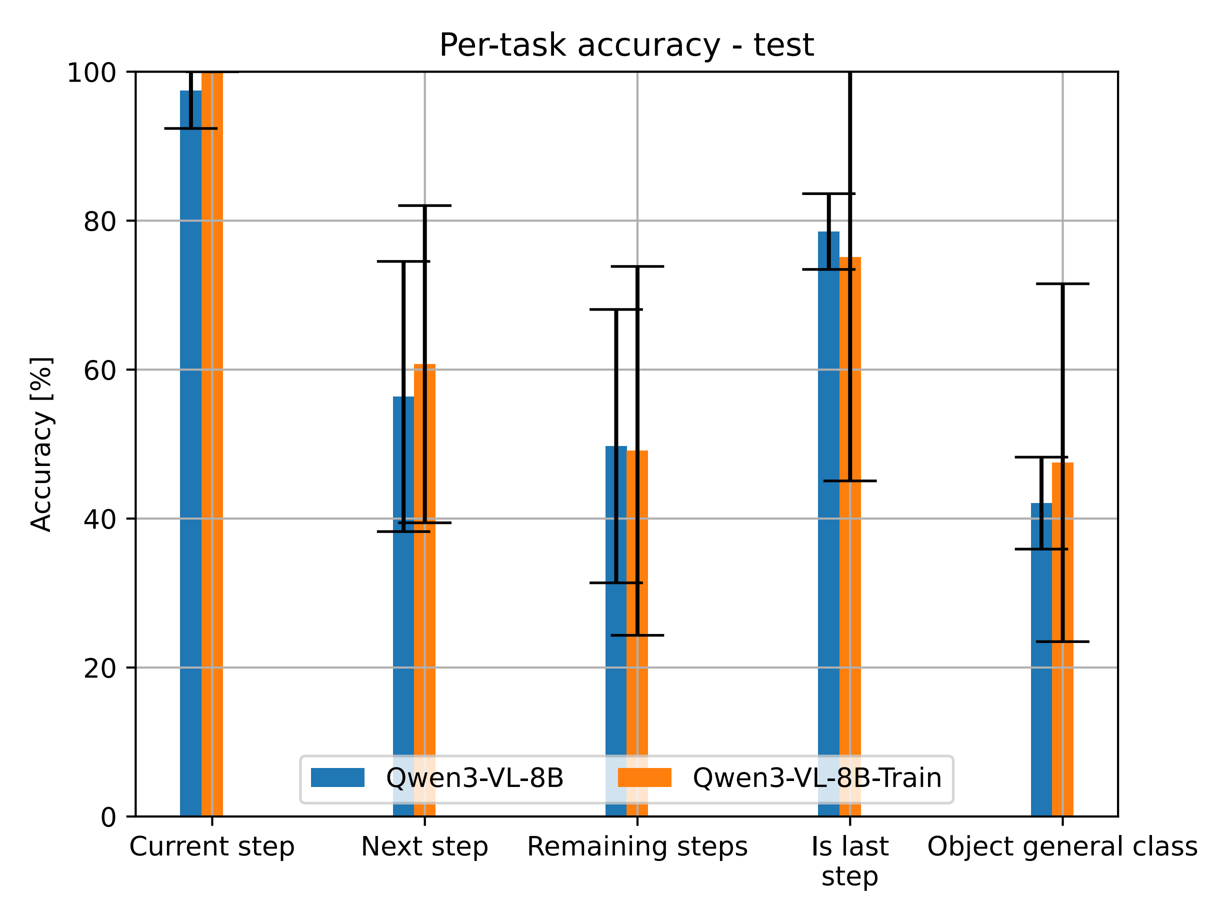

Per-task accuracy - test
Accuracy [%]
0
20
40
60
80
100
Current step
Next step
Remaining steps
Is last step
Object general class
Qwen3-VL-8B
Qwen3-VL-8B-Train
