# Supplementary material for: VLM-guided retrieval augmented generation (RAG) for robot action prediction
Source: Front Neurorobot. 2026 Aug 25;20:1894674. doi: 10.3389/fnbot.2026.1894674 (PMC13550167; doi:10.3389/fnbot.2026.1894674)
Supplement: Supplementary file 1 [file Data_Sheet_1.zip › fig/no_finetune/zero_shot.pdf]

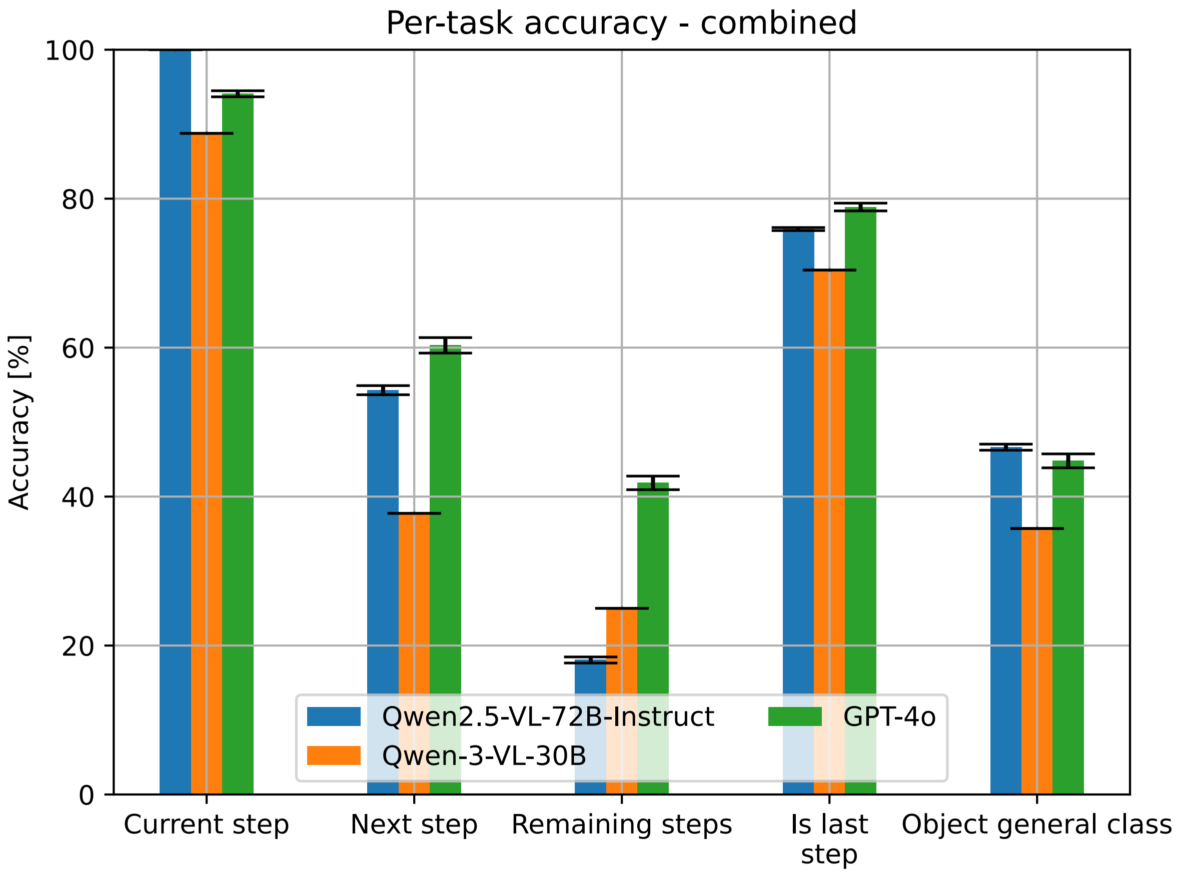
Per-task accuracy - combined
Accuracy [%]
100
80
60
40
20
0
Current step
Next step
Remaining steps
Is last step
Object general class
Qwen2.5-VL-72B-Instruct
Qwen-3-VL-30B
GPT-4o
