# Supplementary material for: VLM-guided retrieval augmented generation (RAG) for robot action prediction
Source: Front Neurorobot. 2026 Aug 25;20:1894674. doi: 10.3389/fnbot.2026.1894674 (PMC13550167; doi:10.3389/fnbot.2026.1894674)
Supplement: Supplementary file 1 [file Data_Sheet_1.zip › fig/no_finetune/single_rag_vs_kg_search.pdf]

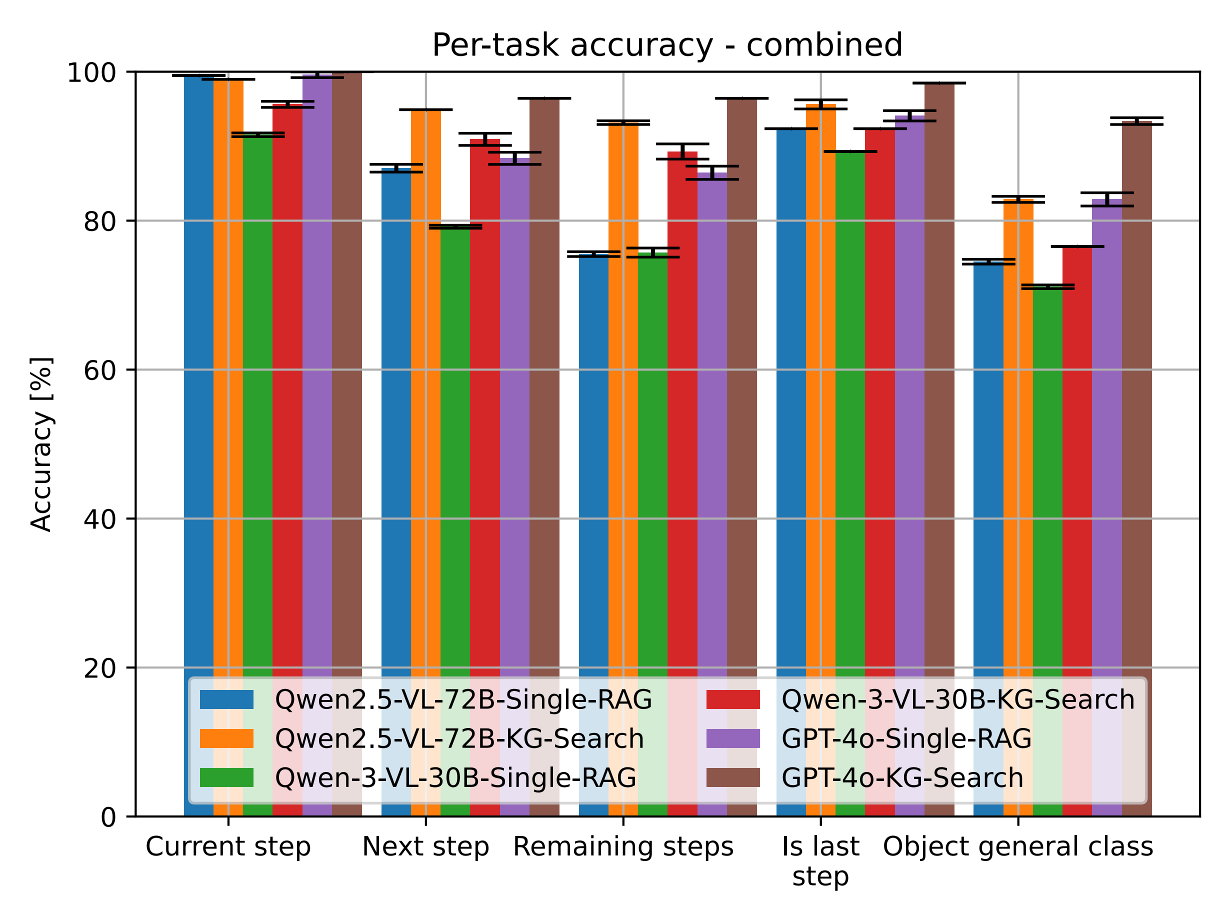

Per-task accuracy - combined
Accuracy [%]
0
20
40
60
80
100
Current step
Next step
Remaining steps
Is last step
Object general class
Qwen2.5-VL-72B-Single-RAG
Qwen2.5-VL-72B-KG-Search
Qwen-3-VL-30B-Single-RAG
Qwen-3-VL-30B-KG-Search
GPT-4o-Single-RAG
GPT-4o-KG-Search
