# Supplementary material for: VLM-guided retrieval augmented generation (RAG) for robot action prediction
Source: Front Neurorobot. 2026 Aug 25;20:1894674. doi: 10.3389/fnbot.2026.1894674 (PMC13550167; doi:10.3389/fnbot.2026.1894674)
Supplement: Supplementary file 1 [file Data_Sheet_1.zip › fig/embedding_model_eval/embedding_lognorm_dist_fit_crop.pdf]

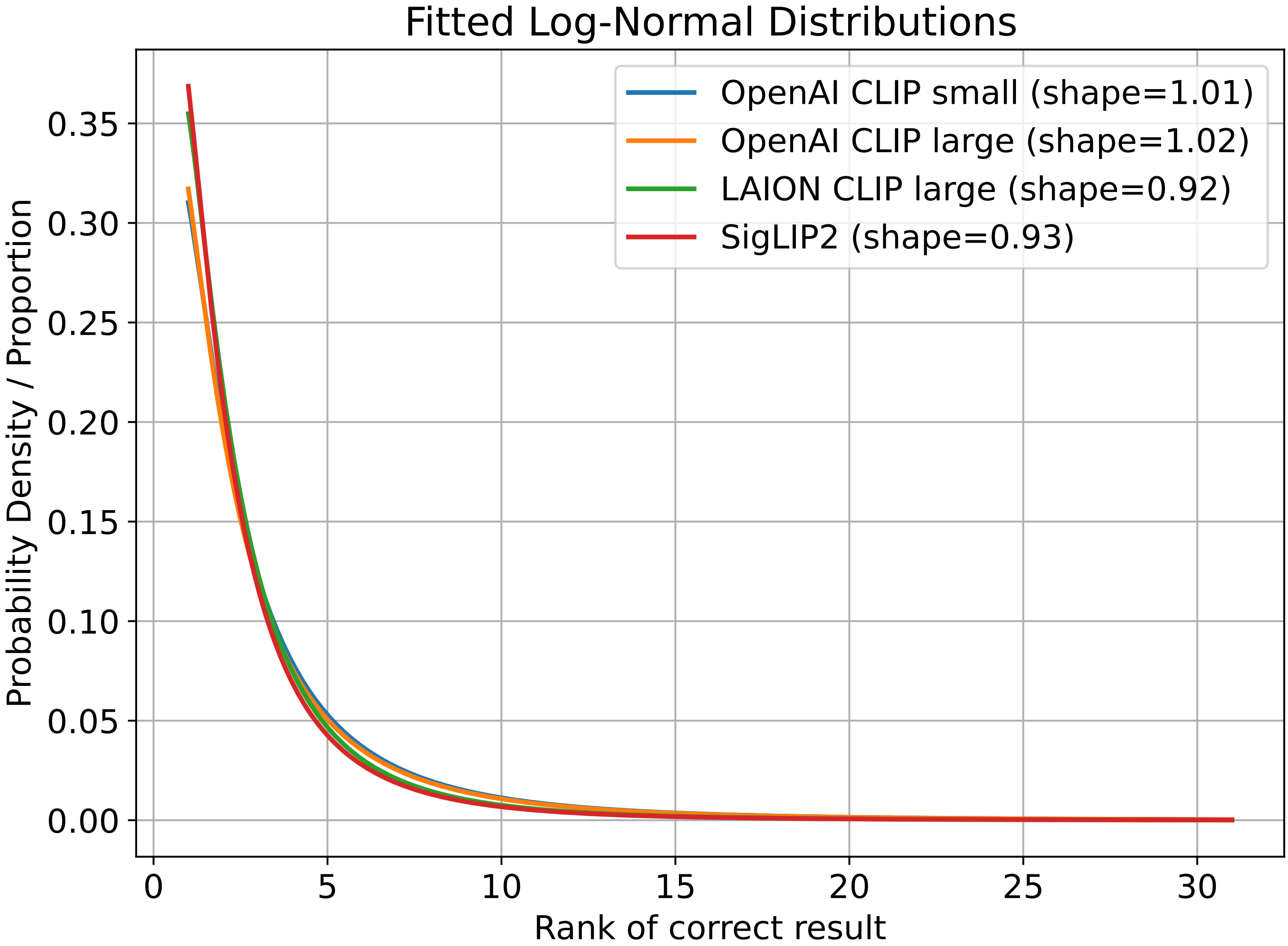
Fitted Log-Normal Distributions
OpenAI CLIP small (shape=1.01)
OpenAI CLIP large (shape=1.02)
LAION CLIP large (shape=0.92)
SigLIP2 (shape=0.93)
Probability Density / Proportion
Rank of correct result
0.00
0.05
0.10
0.15
0.20
0.25
0.30
0.35
0
5
10
15
20
25
30
