# Supplementary material for: VLM-guided retrieval augmented generation (RAG) for robot action prediction
Source: Front Neurorobot. 2026 Aug 25;20:1894674. doi: 10.3389/fnbot.2026.1894674 (PMC13550167; doi:10.3389/fnbot.2026.1894674)
Supplement: Supplementary file 1 [file Data_Sheet_1.zip › fig/knowledge_graph/ActiveRAG-4.pdf]

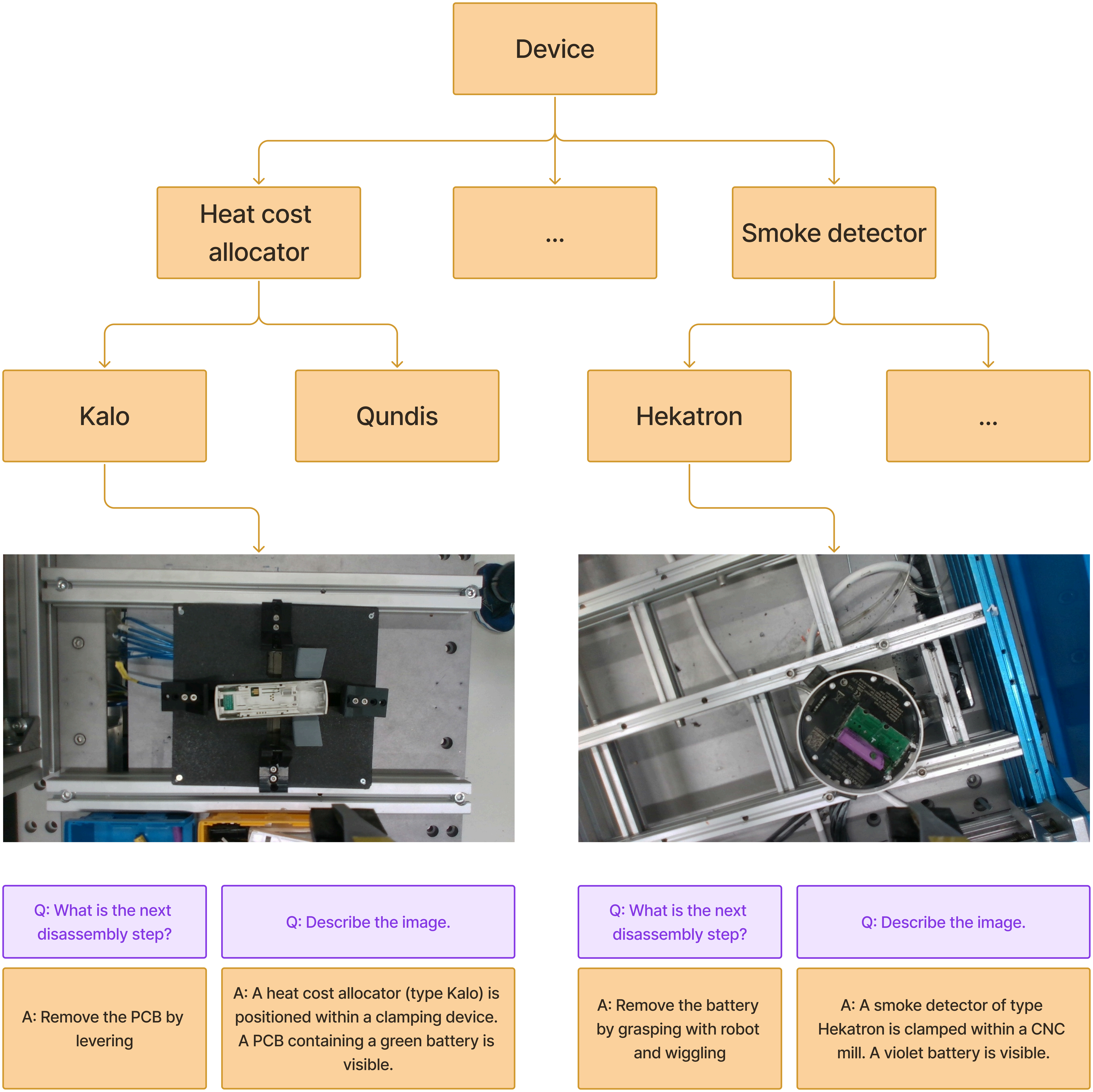
Device
Heat cost allocator
...
Smoke detector
Kalo
Qundis
Hekatron
...
Q: What is the next disassembly step?
Q: Describe the image.
Q: What is the next disassembly step?
Q: Describe the image.
A: Remove the PCB by levering
A: A heat cost allocator (type Kalo) is positioned within a clamping device. A PCB containing a green battery is visible.
A: Remove the battery by grasping with robot and wiggling
A: A smoke detector of type Hekatron is clamped within a CNC mill. A violet battery is visible.
