# Supplementary material for: VLM-guided retrieval augmented generation (RAG) for robot action prediction
Source: Front Neurorobot. 2026 Aug 25;20:1894674. doi: 10.3389/fnbot.2026.1894674 (PMC13550167; doi:10.3389/fnbot.2026.1894674)
Supplement: Supplementary file 1 [file Data_Sheet_1.zip › fig/embedding_model_eval/embedding_dist_hist_noncrop.pdf]

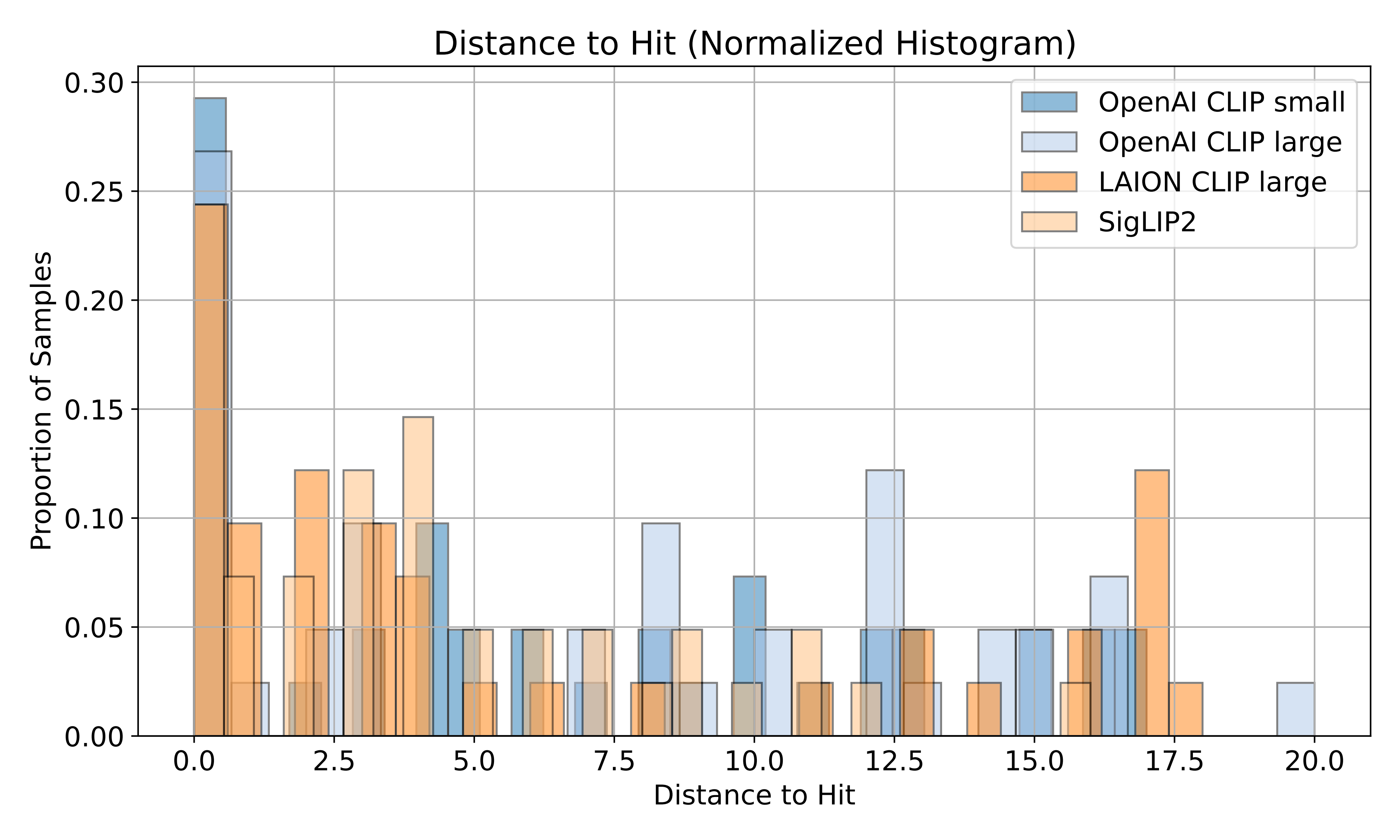

Distance to Hit (Normalized Histogram)
Proportion of Samples
Distance to Hit
OpenAI CLIP small
OpenAI CLIP large
LAION CLIP large
SigLIP2
0.00
0.05
0.10
0.15
0.20
0.25
0.30
0.0
2.5
5.0
7.5
10.0
12.5
15.0
17.5
20.0
