# Supplementary material for: VLM-guided retrieval augmented generation (RAG) for robot action prediction
Source: Front Neurorobot. 2026 Aug 25;20:1894674. doi: 10.3389/fnbot.2026.1894674 (PMC13550167; doi:10.3389/fnbot.2026.1894674)
Supplement: Supplementary file 1 [file Data_Sheet_1.zip › fig/embedding_model_eval/embedding_lognorm_dist_fit_noncrop.pdf]

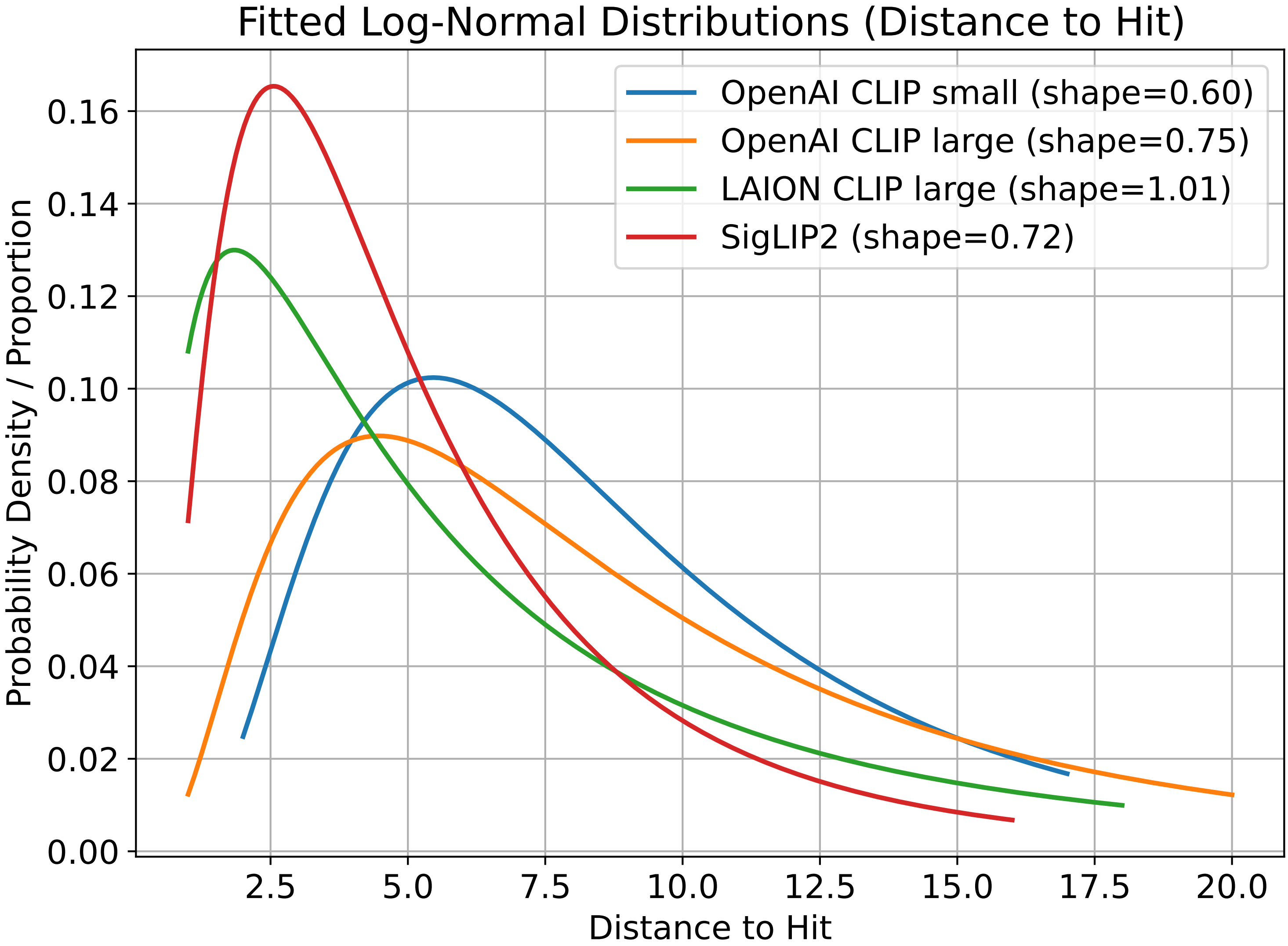

Fitted Log-Normal Distributions (Distance to Hit)
OpenAI CLIP small (shape=0.60)
OpenAI CLIP large (shape=0.75)
LAION CLIP large (shape=1.01)
SigLIP2 (shape=0.72)
Probability Density / Proportion
0.00
0.02
0.04
0.06
0.08
0.10
0.12
0.14
0.16
2.5
5.0
7.5
10.0
12.5
15.0
17.5
20.0
Distance to Hit
