# Supplementary material for: VLM-guided retrieval augmented generation (RAG) for robot action prediction
Source: Front Neurorobot. 2026 Aug 25;20:1894674. doi: 10.3389/fnbot.2026.1894674 (PMC13550167; doi:10.3389/fnbot.2026.1894674)
Supplement: Supplementary file 1 [file Data_Sheet_1.zip › fig/embedding_model_eval/embedding_acc.pdf]

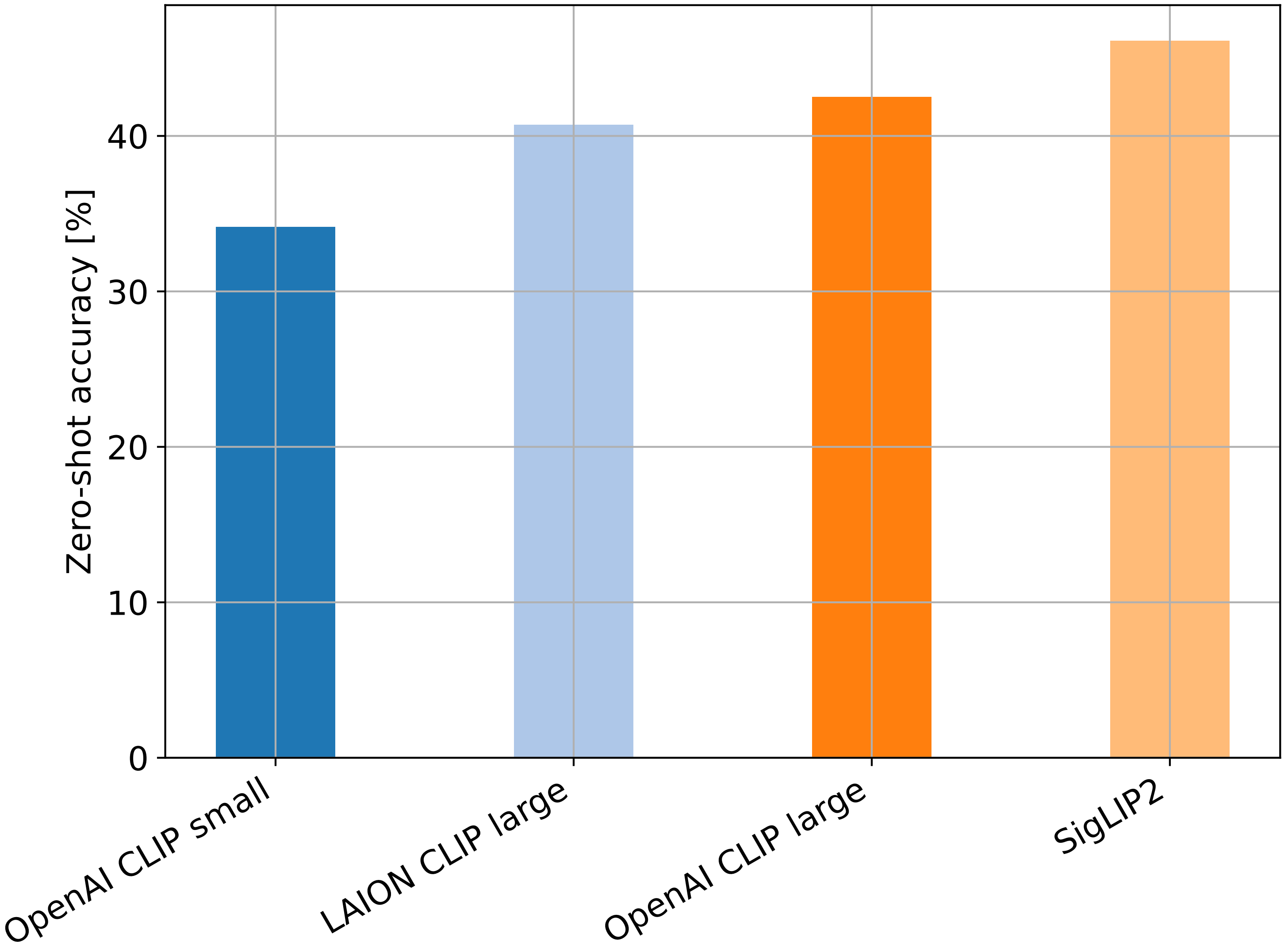
Zero-shot accuracy [%]
0
10
20
30
40
OpenAI CLIP small
LAION CLIP large
OpenAI CLIP large
SigLIP2
