# Supplementary material for: VLM-guided retrieval augmented generation (RAG) for robot action prediction
Source: Front Neurorobot. 2026 Aug 25;20:1894674. doi: 10.3389/fnbot.2026.1894674 (PMC13550167; doi:10.3389/fnbot.2026.1894674)
Supplement: Supplementary file 1 [file Data_Sheet_1.zip › fig/embedding_model_eval/embedding_dist_hist.pdf]

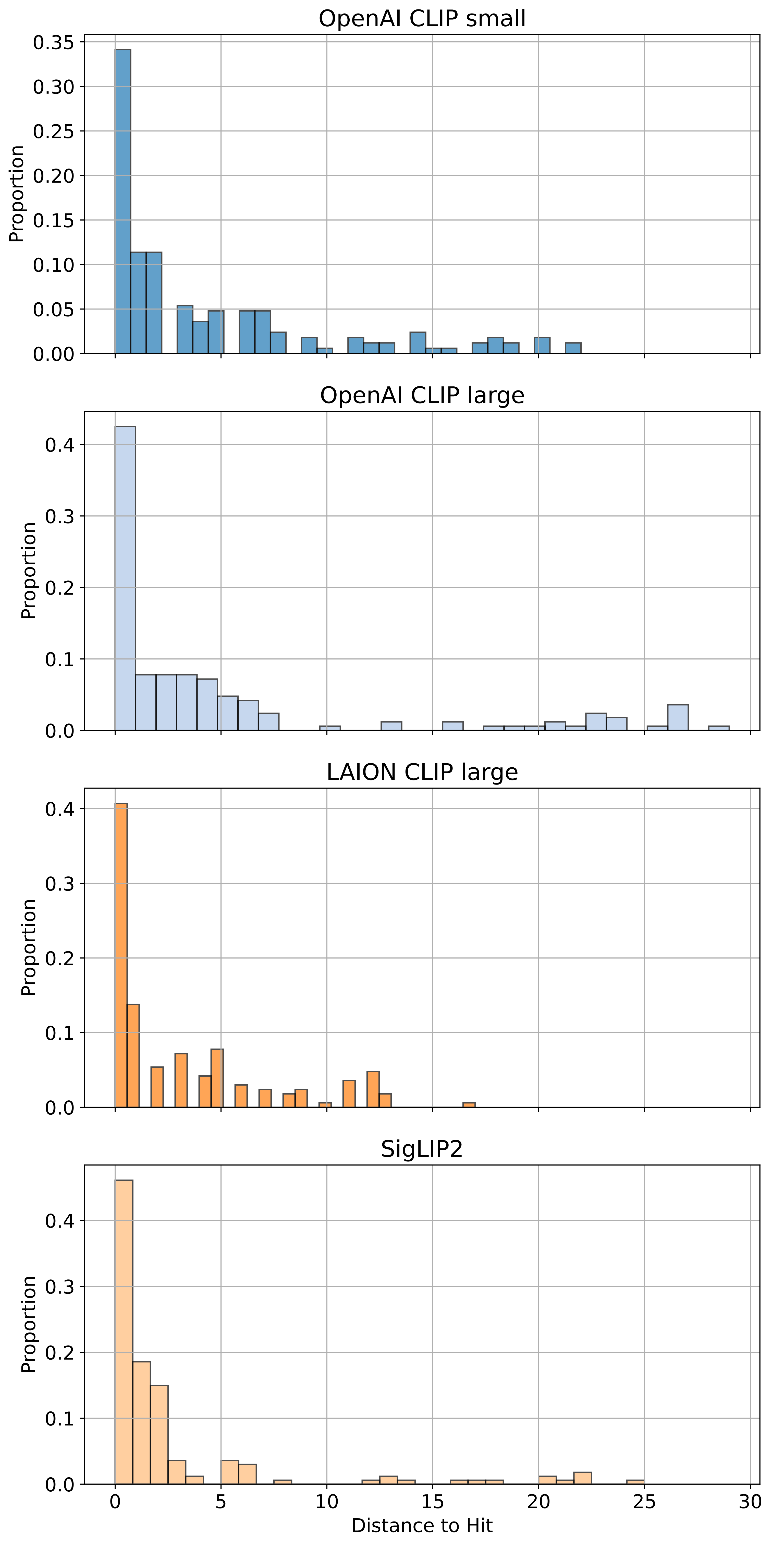

OpenAI CLIP small
OpenAI CLIP large
LAION CLIP large
SigLIP2
Proportion
Distance to Hit
0.00
0.05
0.10
0.15
0.20
0.25
0.30
0.35
0.0
0.1
0.2
0.3
0.4
0
5
10
15
20
25
30
