# Supplementary material for: VLM-guided retrieval augmented generation (RAG) for robot action prediction
Source: Front Neurorobot. 2026 Aug 25;20:1894674. doi: 10.3389/fnbot.2026.1894674 (PMC13550167; doi:10.3389/fnbot.2026.1894674)
Supplement: Supplementary file 1 [file Data_Sheet_1.zip › fig/embedding_model_eval/embedding_exp_dist_fit.pdf]

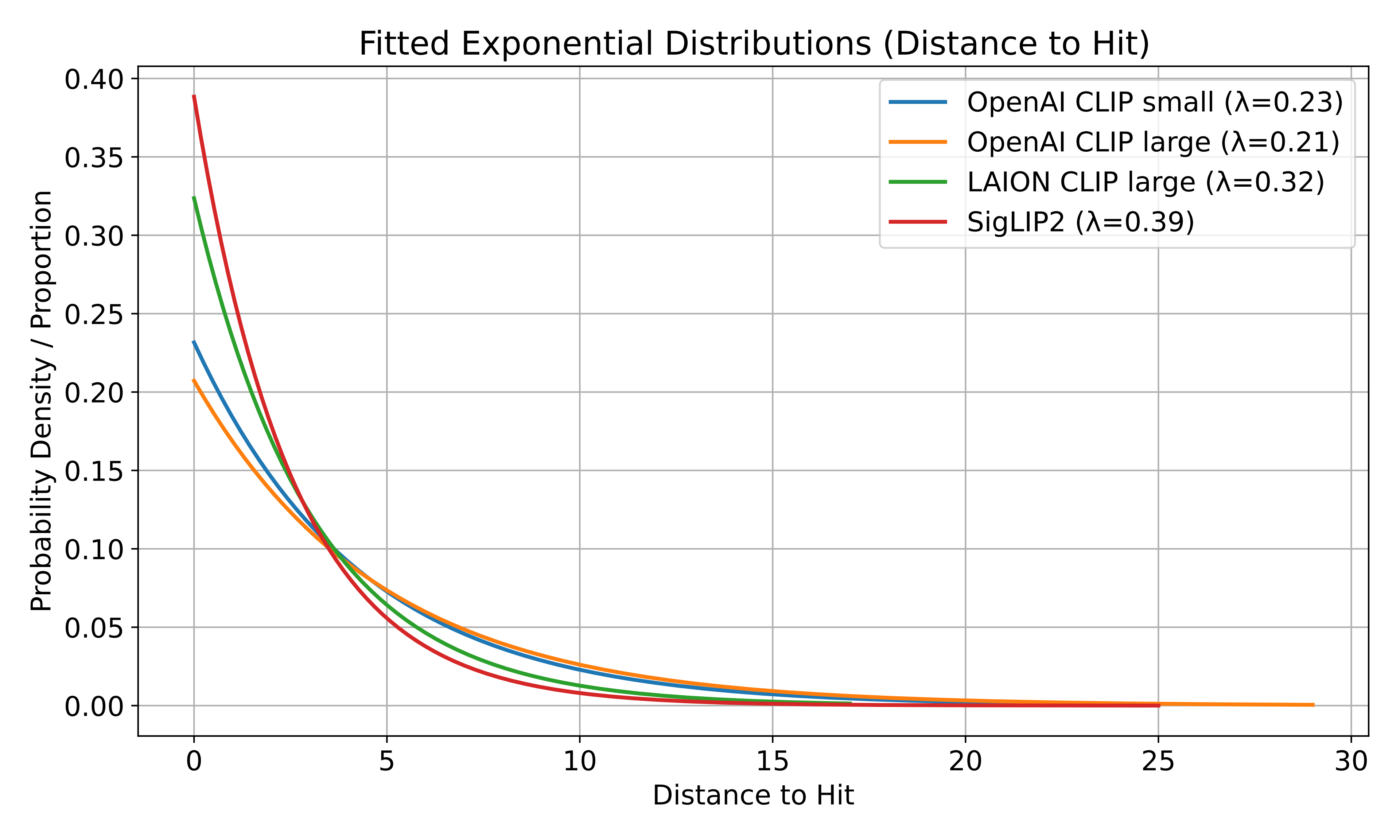
Fitted Exponential Distributions (Distance to Hit)
OpenAI CLIP small (λ=0.23)
OpenAI CLIP large (λ=0.21)
LAION CLIP large (λ=0.32)
SigLIP2 (λ=0.39)
Probability Density / Proportion
0.00
0.05
0.10
0.15
0.20
0.25
0.30
0.35
0.40
0
5
10
15
20
25
30
Distance to Hit
